# Pan-cancer analysis identifies proteasome 26S subunit, ATPase (PSMC) family genes, and related signatures associated with prognosis, immune profile, and therapeutic response in lung adenocarcinoma

**DOI:** 10.3389/fgene.2022.1017866

**Published:** 2023-01-09

**Authors:** Hui Jia, Wen-Jin Tang, Lei Sun, Chong Wan, Yun Zhou, Wei-Zhong Shen

**Affiliations:** ^1^ Department of Thoracic Surgery, Jiangsu Cancer Hospital, Jiangsu Institute of Cancer Research, The Affiliated Cancer Hospital of Nanjing Medical University, Nanjing, China; ^2^ Department of Nursing, Jiangsu Cancer Hospital, Jiangsu Institute of Cancer Research, The Affiliated Cancer Hospital of Nanjing Medical University, Nanjing, China; ^3^ Department of Interventional Radiology, Jiangsu Cancer Hospital, Jiangsu Institute of Cancer Research, The Affiliated Cancer Hospital of Nanjing Medical University, Nanjing, China; ^4^ Yangtze Delta Region Institute of Tsinghua University, Jiaxing, China; ^5^ Department of Medical Oncology, Jiangsu Cancer Hospital, Jiangsu Institute of Cancer Research, The Affiliated Cancer Hospital of Nanjing Medical University, Nanjing, China

**Keywords:** PSMC, LUAD, prognosis, immune, immunotherapy, chemotherapy, CD276

## Abstract

**Background:** Proteasome 26S subunit, ATPase gene (PSMC) family members play a critical role in regulating protein degradation and are essential for tumor development. However, little is known about the integrative function and prognostic significance of the PSMC gene family members in lung cancer.

**Methods:** First, we assessed the expression and prognostic features of six PSMC family members in pan-cancer from The Cancer Genome Atlas (TCGA) dataset. Hence, by focusing on the relationship between PSMC genes and the prognostic, genomic, and tumor microenvironment features in lung adenocarcinoma (LUAD), a PSMC-based prognostic signature was established using consensus clustering and multiple machine learning algorithms, including the least absolute shrinkage and selection operator (LASSO) Cox regression, CoxBoost, and survival random forest analysis in TCGA and GSE72094. We then validated it in three independent cohorts from GEO and estimated the correlation between risk score and clinical features: genomic features (alterations, tumor mutation burden, and copy number variants), immune profiles (immune score, TIDE score, tumor-infiltrated immune cells, and immune checkpoints), sensitivity to chemotherapy (GDSC, GSE42127, and GSE14814), and immunotherapy (IMvigor210, GSE63557, and immunophenoscore). Twenty-one patients with LUAD were included in our local cohort, and tumor samples were submitted for evaluation of risk gene and PD-L1 expression.

**Results:** Nearly all six PSMC genes were overexpressed in pan-cancer tumor tissues; however, in LUAD alone, they were all significantly correlated with overall survival. Notably, they all shared a positive association with increased TMB, TIDE score, expression of immune checkpoints (CD276 and PVR), and more M1 macrophages but decreased B-cell abundance. A PSMC-based prognostic signature was established based on five hub genes derived from the differential expression clusters of PSMC genes, and it was used to dichotomize LUAD patients into high- and low-risk groups according to the median risk score. The area under the curve (AUC) values for predicting survival at 1, 3, and 5 years in the training cohorts were all >.71, and the predictive accuracy was also robust and stable in the GSE72094, GSE31210, and GSE13213 datasets. The risk score was significantly correlated with advanced tumor, lymph node, and neoplasm disease stages as an independent risk factor for LUAD. Furthermore, the risk score shared a similar genomic and immune feature as PSMC genes, and high-risk tumors exhibited significant genomic and chromosomal instability, a higher TIDE score but lower immune score, and a decreased abundance of B and CD8^+^ T cells. Finally, high-risk patients were suggested to be less sensitive to immunotherapy but had a higher possibility of responding to platinum-based chemotherapy. The LUAD samples from the local cohort supported the difference in the expression levels of these five hub genes between tumor and normal tissues and the correlation between the risk score and PD-L1 expression.

**Conclusion:** Overall, our results provide deep insight into PSMC genes in LUAD, especially the prognostic effect and related immune profile that may predict therapeutic responses.

## 1 Introduction

Lung cancer is the most prevalent cancer and a prominent cause of cancer-related mortality worldwide (Thai et al., 2021). Non-small-cell lung cancer (NSCLC) accounts for 85% of all lung cancers, with lung adenocarcinoma (LUAD) being the most common subtype. Despite great advances in the era of precision oncology, the 5-year survival of patients with LUAD remains unsatisfactory (Miller and Hanna, 2021). Meanwhile, LUAD is a disease with great heterogeneity, which results in different outcomes for patients treated with the same regimens (Memmott et al., 2021). Therefore, there is a practical need to develop accurate and promising prognostic biomarkers and efficient therapeutic targets for clinicians to tailor the most appropriate treatment to prolong the survival of patients with LUAD.

Dysfunctional proteasomes correlate with cancer by disrupting the degradation of proteins involved in regulating cell growth or death (Mani and Gelmann, 2005). Protein degradation by the proteasome is important for inflammation and antigen presentation in cancer and has been associated with the response to immune checkpoint inhibitors (ICIs) (Kalaora et al., 2020). The proteasome 26S subunit, ATPase gene (PSMC) family consists of six members, including PSMC1–6, which are essential components of the 19S regulatory particle of the proteasome (Kamber Kaya and Radhakrishnan, 2020). In addition, previous studies have revealed that each of the six PSMC family members is crucial for carcinogenesis and progression of different cancer types (Zhang Y. et al., 2020; Wang et al., 2021). For instance, PSMC2, the most investigated one, has been found to be upregulated in various types of cancer, including gastric cancer (Liu et al., 2022), ovarian cancer (Zhu et al., 2021), and hepatocellular carcinoma (Duan et al., 2021a), promoting tumor cell proliferation and invasion. Similarly, with fewer studies, other PSMC genes have been identified as sole oncogenes in certain cancers (Xiaohang et al., 2018; Zhou et al., 2020; He et al., 2021). The regulation of cancer cell development by PSMC genes has been studied in multiple cancers but rarely in LUAD, and their function as oncogenes was found to be correlated with dysfunctions in the cell cycle (Liu Y. et al., 2021), PI3K/AKT/mTOR (Zhang Y. et al., 2020; Wang et al., 2022), MAPK (Jang et al., 2015), and EMT pathways (He et al., 2021). Furthermore, PSMC family genes have widely proved their function in the regulation of cancer treatment. By using a CRISPR experiment targeted at 19,052 genes, Shi et al. found that PSMC6 was the only gene significantly associated with bortezomib resistance in myeloma cells (Shi et al., 2017). The prevalence of PSMC family gene genomic alterations in pan-cancers ranges from 1.1% to 2.5%, with amplification being the most common alteration type (TCGA PanCancer Atlas Studies, www.cbioportal.org). Therefore, assessing their expression levels is more reasonable.

Given that all six PSMC proteins form the foundation of the 19S regulatory particle together, unfolding and translocating the substrate (Rousseau and Bertolotti, 2018), it is more reasonable to examine PSMC genes collaboratively. However, the relationship between PSMC family members and the prognosis of patients with LUAD remain unclear. Moreover, even though they are not pivotal modulators that switch constitutive proteasomes to immunoproteasomes, it is still unclear whether and how they would shape tumor immunity and influence the response to ICIs in LUAD. Recently, Md. Asad Ullah and his colleagues have uncovered the genomic mutation, expression, and methylation characteristics of PSMC genes (PSMC1–5, but missing PSMC6) in LUAD (Ullah et al., 2022). Even though they shed light on the weak correlation between the expression level of PSMC genes and IDO1 and CD274, more comprehensive and in-depth research is required to determine the PSMC family genes’ biological function and whether they can be used as effective prognostication biomarkers for therapeutic selection.

In this study, we hypothesized that PSMC family genes are significant and that their expression may provide prognostic prediction and therapeutic guidance for patients with LUAD. Based on the transcriptomic profile of The Cancer Genome Atlas (TCGA) and multiple other datasets, the expression patterns of PSMC and related clinical, genomic, and tumor immune features were investigated, and a robust and stable prognostic model was developed. The results of this study are expected to yield a comprehensive understanding of PSMC genes in LUAD and an improved predictive stratification tool with treatment indications for patients with LUAD.

## 2 Materials and methods

### 2.1 Study design

A schematic diagram of the study design is presented in Supplementary Figure S1.

### 2.2 Collection of LUAD datasets

RNA sequencing (RNA-seq) data and information on clinicopathological features of patients with LUAD were obtained from TCGA database (https://portal.gdc.cancer.gov) as the training set. External validation datasets, including GSE72094 (*n* = 398), GSE31210 (*n* = 226), and GSE13213 (*n* = 117), were obtained from the GEO database (https://www.ncbi.nlm.nih.gov/geo/).

### 2.3 Comparison of PSMC gene expression between tumor and normal tissues

Analysis of the difference in the expression level of the six PSMC genes between tumor and normal control samples in pan-cancer was performed by using the GEPIA database (www.gepia.cancer-pku.cn/index.html) (Zefang et al., 2017).

### 2.4 Development of the PSMC gene family prognostic signature

First, consensus clustering was performed based on the expression pattern of PSMC family genes in the TCGA and GSE72094 datasets by using the R package “ConsensusClusterPlus.” The optimal number of clusters was then selected based on an inspection of the Delta CDF (change data feed) plot in these two cohorts. Kaplan–Meier survival analysis was performed to assess the differences in survival between clusters. Differentially expressed genes (DEGs) between clusters in TCGA or GSE72094 datasets were determined by using the R package “LIMMA” (version 3.50.3), and the FDR-adjusted *p*-value of <.000001 was set as the threshold to select DEGs. After screening out the overlapped cluster-related DEGs between TCGA and GSE72094 datasets, three machine learning algorithms were used to select candidate genes. Least absolute shrinkage and selection operator (LASSO) Cox was performed using the R package “glmnet” (version 4.1-4) with 10-fold cross validation, and lambda.min () was chosen to select the best model. In the interim, the R package “CoxBoost” (version 1.5) was used to perform CoxBoost analysis with 10-fold cross validation. The optimal boosting steps were optimized using the optimCoxBoostPenalty method. Survival random forest was then performed using the R package “randomForestSRC” (version 4.1-4). Finally, the overlapping candidate genes selected by all three algorithms were applied to develop a risk signature. The minimum criteria were used to confirm the penalty parameter (λ), and the coefficients of the six genes were retained. The risk score formula is as follows: risk score = the expression level of gene A × γA + the expression level of gene B × γB +…+ the expression level of gene Z × γZ, where γ indicates the coefficients. Based on the median risk score, the patients with LUAD were classified into low- and high-risk groups. Kaplan–Meier analysis was used to analyze the overall survival (OS) of patients in the low- and high-risk groups. The “timeROC” package in R Studio was used to calculate the area under the receiver operating characteristic (ROC) curve (AUC), which is an important standard for evaluating the prognostic ability of the PSMC signature. According to the same formula, patients from the GSE72094, GSE31210, and GSE13213 cohorts were dichotomized into low- and high-risk groups based on the median risk score in each cohort. Kaplan–Meier and ROC analyses were performed to evaluate the performance of survival prediction at 1 to 5 years.

### 2.5 Comparison of predictive performance between the established signature and previously reported signatures in LUAD

The prediction accuracy for survival between the established PSMC signature and seven other risk signatures (Zhang C. et al., 2020; Al-Dherasi et al., 2021; Liu L-P. et al., 2021; Fan et al., 2021; Li et al., 2022; Zhang et al., 2022; Zhu et al., 2022) was compared. The risk score for each signature was calculated based on the formula retrieved from the articles; these formulas are presented in Supplementary Table S1.

### 2.6 Nomogram construction

A nomogram was constructed by combining the risk score and clinicopathological parameters for the prediction of 1-, 3-, and 5-year survival in patients with LUAD. The model’s discrimination performance was assessed using AUC, and the R package “RMS” was used to evaluate the heterogeneities in predicting the ability of the model.

### 2.7 Genomic profile analysis

Genomic alterations in each sample in TCGA cohort were analyzed using the R package “Maftools.” In the meantime, the R package “GISTIC2.0” was used to analyze and visualize the copy number variants (CNVs) in the genomic regions of each tumor sample with the default parameters. Gain and loss in copy number were identified using the default GISTIC threshold. Actionable alterations in the TCGA-LUAD cohorts were identified using the OncoKB database (https://www.oncokb.org/#/). Then, the difference in the frequency of gene alterations or CNV between the groups was compared using Fisher’s exact test.

### 2.8 Tumor immune microenvironment

To probe the correlation between the risk score and tumor immune microenvironment (TME), the ESTIMATE algorithm was used to determine the immune, stromal, and ESTIMATE scores of each patient in TCGA cohort. The differences in the immune, stromal, and ESTIMATE scores between the high- and low-risk groups were evaluated using the Wilcoxon test. The CIBERSORT algorithm according to the “CIBERSORT” R package (CIBERSORT R script v1.03; http://cibersort.stanford.edu/) was used to calculate the proportion of 22 tumor-infiltrated lymphocytes (TILs) in the tumor microenvironment. Other algorithms, including xCell (Aran et al., 2017), TIMER (Li et al., 2020), MCP-counter (Becht et al., 2016), TIP (Xu et al., 2018), quanTIseq (Finotello et al., 2019), and EPIC (Racle et al., 2017), were also used to comprehensively evaluate TIL levels in each tumor sample.

### 2.9 Calculation of T-cell dysfunction and exclusion score

The T-cell dysfunction and exclusion (TIDE) score of each sample was analyzed through the TIDE website (http://tide.dfci.harvard.edu/login/) to predict the level of T-cell dysfunction and inhibition of T-cell infiltration (Jiang et al., 2018). This computational method has the capacity to predict the response of ICIs.

### 2.10 Assessment of the sensitivity to immunotherapy

The association between the PSMC risk score and the response to atezolizumab (an anti-PD-L1 blockade) was evaluated in the IMvigor210 cohort using the R package “IMvigor210CoreBiologies.” Transcriptomic, survival, and therapeutic response data (including durable clinical benefit [DCB], complete response [CR], partial response [PR], stable disease [SD], progressive disease [PD]) were retrieved from the dataset. Meanwhile, the GSE63557 dataset containing AB1-HA mesothelioma mice treated with anti-cytotoxic T-lymphocyte-associated protein 4 (CTLA4) blockade was used to investigate the difference in the risk score between responders and non-responders (Lesterhuis et al., 2015). Immunophenoscore (IPS) is an algorithm used to assess the immunophenotype of a tumor sample by integrating the levels of MHC molecules, immunomodulator effector cells, and suppressor cells, which is positively correlated with enhanced immunogenicity (Charoentong et al., 2017). Each sample from TCGA-LUAD cohort was analyzed using the Cancer Immune Group Atlas (TCIA, https://tcia.at/home) website.

### 2.11 Evaluation of chemotherapy response

First, the sensitivity of LUAD patients to chemotherapeutic drugs, including docetaxel, paclitaxel, vinblastine, gemcitabine, cisplatin, erlotinib, gefitinib, doxorubicin, and etoposide, was predicted using the Genomics of Drug Sensitivity in Cancer (GDSC; https://www.cancerrxgene.org) database. The half maximal inhibitory concentration (IC50) of each drug in an individual sample was determined using the R package “pRRophetic.” Subsequently, the transcriptomic and clinical data of patients with LUAD receiving adjuvant chemotherapy or not were retrieved from the GSE42127 (mainly treated with carboplatin plus taxanes) and GSE14814 datasets (cisplatin/vinorelbine). Only LUAD samples were retained for further analysis in both datasets.

### 2.12 Real-time polymerase chain reaction

Twenty-one patients with LUAD were included in our local cohort, and tumor samples were submitted for evaluation of the risk gene and PD-L1 expression. The study protocol was approved by the ethical committee of Jiangsu Cancer Hospital (approval no. 2021-090-01), and all participants provided informed consent. Patient clinical data are presented in Supplementary Table S2. Total RNA was extracted from the collected tissues using the TRIzol reagent (Invitrogen, CA, United States), and cDNA was synthesized using a high-capacity cDNA reverse transcription kit (Thermo Fisher Scientific, CA, United States) according to the manufacturer’s protocol. Real-time PCR was performed to detect the expression of GNPNAT1, LDHA, SEC61G, PLEK2, and C1QTNF6 using PowerUp SYBR Green Master Mix (Thermo Fisher Scientific, CA, United States). All samples were analyzed using an ABI 7900HT PCR machine (Thermo Fisher, CA, United States). The gene expression levels were normalized to those of GAPDH. Primer sequences for the genes were as follows: GNPNAT1 (forward primer: 5′-ACT​CCT​ATG​TTT​GAC​CCA​AGT​CT-3′, reverse primer: 5′-TCT​GTT​AGC​TGA​CCC​AAT​ACC​T-3′); LDHA (forward primer: 5′-ATG​GCA​ACT​CTA​AAG​GAT​CAG​C-3′, reverse primer: 5′-CCA​ACC​CCA​ACA​ACT​GTA​ATC​T-3′); SEC61G (forward primer: 5′-GCA​GTT​TGT​TGA​GCC​AAG​TCG-3′, reverse primer: 5′-CCA​GCC​GAA​TGG​AGT​CCT​T-3′); PLEK2 (forward primer: 5′-GCG​ATG​GTT​CAT​CCT​TCG​G-3′, reverse primer: 5′-ATA​GCC​CCG​GTG​ATC​TCA​AAG-3′); C1QTNF6 (forward primer: 5′-TGC​CTG​AGA​TCA​GAC​CCT​ACA, reverse primer: 5′-GCC​CAC​TGA​GAA​GGC​GAA​G-3′); and GAPDH (forward primer: 5′-CTG​GGC​TAC​ACT​GAG​CAC​C-3′, reverse primer: 5′-AAG​TGG​TCG​TTG​AGG​GCA​ATG -3′).

### 2.13 PD-L1 staining

PD-L1 status was assessed using an anti-PD-L1 antibody (22C3) (Agilent, CA, United States), and all stained slides for PD-L1 membrane staining were reviewed by two independent pathologists. The tumor proportion score (TPS) is defined as the percentage of viable tumor cells showing partial or complete membrane staining relative to all viable tumor cells present in the sample, and samples with TPS ≥1% were classified as PD-L1 positive (Reck et al., 2016).

### 2.14 Statistical analysis

The patients were divided into the high- and low-risk groups using the median cutoff of the expression level or the risk score. Comparisons in clinical demographics between two groups were assessed with Student’s t-test or the Wilcoxon test. Student’s t-test or the Wilcoxon test was used to compare clinical demographics between samples with high expression and low expression and between groups with high and low risk scores. Differences in OS were compared between groups with Kaplan–Meier curves, Cox regression, and log-rank tests. The correlation between the PSMC gene or risk score and other variables was conducted by Spearman’s correlation analysis. The time-dependent area under the receiver operating characteristic curve (AUC) was used to evaluate the predictive power of the risk score on OS, and a higher value of AUC represented higher accuracy. The statistical difference in distribution in three or more groups was examined by the Kruskal–Wallis test and that of two groups was compared by the Wilcoxon test. All statistical significances were determined using a two-sided p0.05 cutoff. All statistical analyses were performed using R software (version 4.0.5), the Bioconductor (http://bioconductor.org/) package, and GraphPad Prism 9.0 (GraphPad Software).

## 3 Results

### 3.1 Expression and correlation of PSMC genes with survival in pan-cancer

**FIGURE 1:**
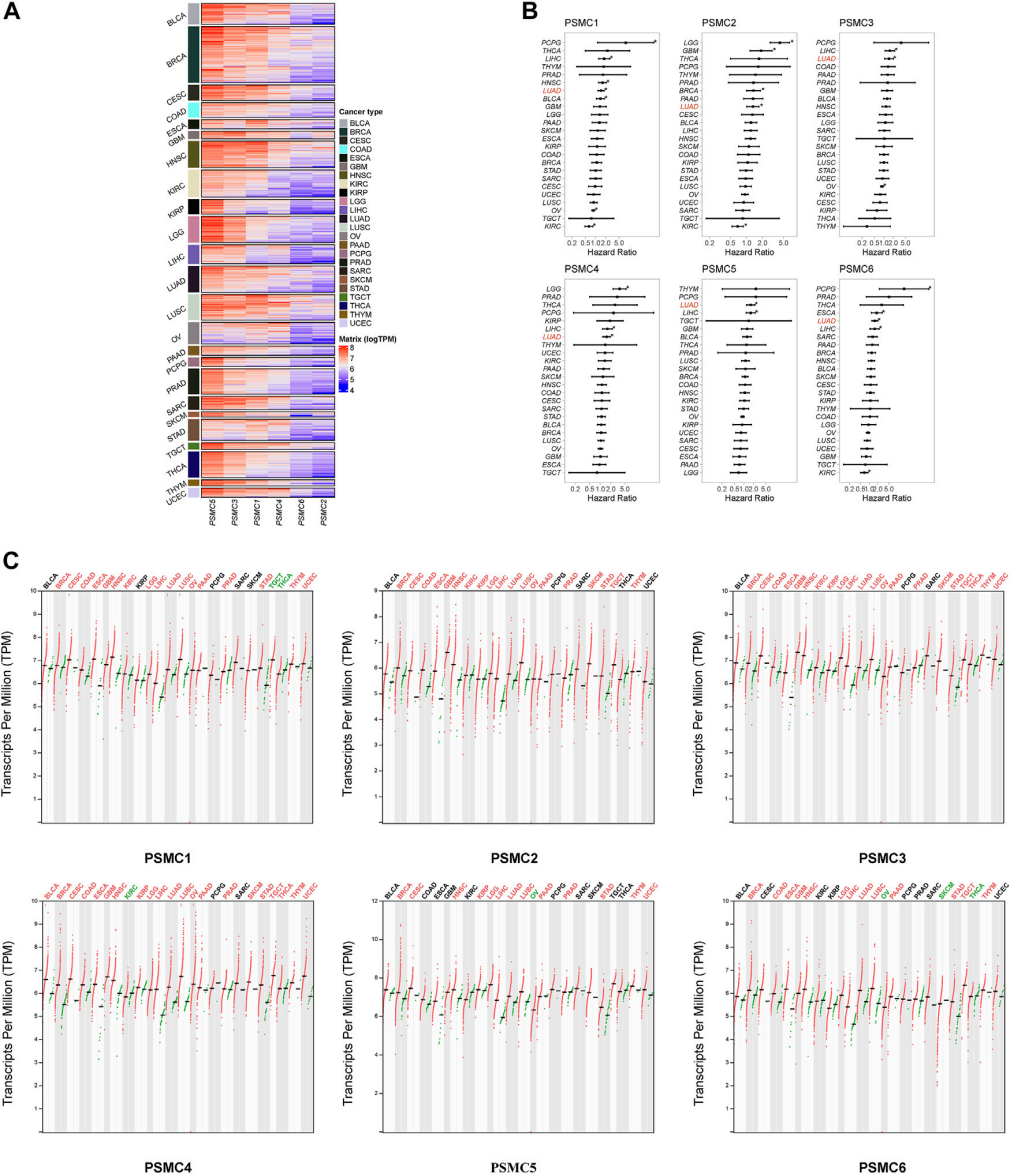
Expression of proteasome 26S subunit, ATPase (PSMC) genes across different types of cancer. **(A)** Heatmap representing the expression level of the six PSMC family genes in pan-cancer. **(B)** Forest plots illustrating the hazard ratio of the expression levels of PSMC genes related to overall survival using univariate Cox analysis. **(C)** Comparison of the expression levels of the six PSMC family genes between tumor and normal tissues in 24 types of cancer. Cancer types labeled black indicated no significant difference between tumor and normal tissues; red indicated that the expression level was significantly upregulated in the tumor tissues; and green indicated that the expression level was significantly downregulated in the tumor tissues.

First, we evaluated the expression levels of the six PSMC family members in 24 types of cancer in TCGA database and found that PSMC5 was the most predominant in all types of cancer except ovarian cancer, whereas PSMC2 and PSMC6 had a relatively lower expression level in pan-cancer (Figure 1A). Using univariate Cox analysis, the relationship between the expression levels of the six PSMC family members and OS was investigated. Notably, only in LUAD, the expression levels of all six PSMC family members were substantially linked with inferior OS (Figure 1B). In addition, the expression levels of all six PSMC family genes were significantly different between tumor and normal tissues in the majority of these 24 cancers, particularly LUAD (Figure 1C). Thus, we focused on studying the integrative role of PSMC family genes in LUAD based on these preceding data. PSMC family genes were rarely mutated in pan-cancers, with amplification as the predominant mutational type (Supplementary Figure S2A); in LUAD, their alterations were likewise uncommon (Supplementary Figure S2B). Then, we analyzed the DNA methylation pattern of PSMC family genes in LUAD (Supplementary Figure S2C), which revealed great heterogeneity between patients. Commonly but slightly, the methylation levels of CpG(s) were found to be negatively correlated with the mRNA expression level of PSMC family genes (Supplementary Figure S2D). The methylation of CpG(s) was not significantly associated with the OS of LUAD patients, with the only exception of cg17757000 (PSMC4), cg10840864, and cg07117700 (PSMC5, Supplementary Figure S2E). As mRNA expression of PSMC genes was more prevalent than methylation and genomic alterations and their expression level was more significantly related to the prognosis of LUAD patients, we focused on their expression level in the subsequent study.

### 3.2 Correlation between PSMC genes and clinical, genomic, and TME features in LUAD

**FIGURE 2:**
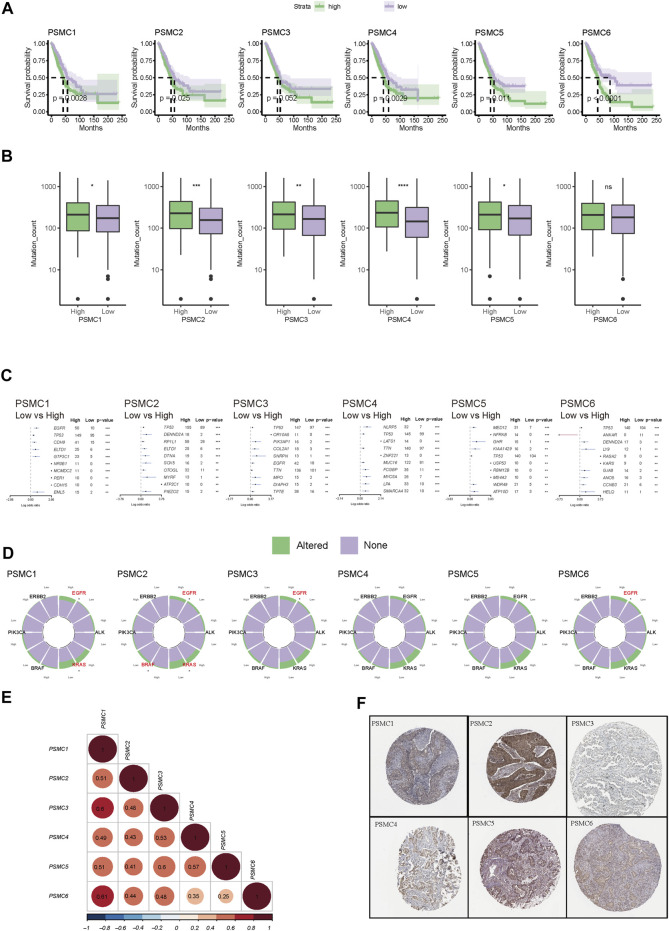
Correlation between PSMC gene expression and the prognostic and genomic features in lung adenocarcinoma (LUAD). **(A)** Kaplan–Meier curves stratified by the expression level of each PSMC gene family member. Patients were classified into high- or low-expression groups based on the median expression cutoff value of each PSMC gene. **(B)** Differences in the mutation counts between the high- and low-expression groups of each PSMC gene. **(C)** Genomic difference between the high- and low-expression groups of each PSMC gene. Those genes with log (odds ratio) over 0 were more prevalent at the high-expression level. **(D)** Differences in the genomic alterations of driver genes, including EGFR, ALK (translocation), KRAS, BRAF, PIK3CA, and ERBB2. Also, those genes labeled with the red color had a significant difference in prevalence between high and low expression levels. **(E)** Expression correlation between each PSMC gene. **(F)** Protein expression of each PSMC gene in LUAD revealed by immunochemistry in the Human Protein Atlas. *p < .05, **p < .01, ***p < .001, and ****p < .0001.

According to the findings of the pan-cancer analysis, all six PSMC genes were identified as oncogenes in LUAD, since patients with overexpression of each PSMC gene had a shorter OS (Figure 2A). Across all six PSMC genes, only PSMC2 and PSMC4 exhibited a significant correlation with lymph node metastasis stage and neoplasm disease stage (*p* < .05, Supplementary Figure S3A), and groups with PSMC2 and PSMC4 overexpression had a higher number of patients with advanced lymph node metastasis and neoplasm disease stages. Additionally, LUAD samples with advanced neoplasm disease stage had increasing expression level of the PSMC gene (Supplementary Figure S3B). Except for PSMC6, groups with overexpression of the other five PSMC genes had significantly higher levels of mutation counts than those with low expression levels (Figure 2B). Intriguingly, all PSMC genes displayed a weak but significant positive correlation with mutation counts (Supplementary Figure S3C), which may account for the fact that nearly all genes with a significantly higher prevalence were present in the overexpression groups (Figure 2C). Subsequently, we investigated the actionable genomic alterations associated with PSMC genes and found that *EGFR* was significantly more prevalent in samples overexpressing PSMC genes, with the exception of PSMC4 and PSMC5 (Figure 2D). As a gene known to be mutually exclusive of *EGFR*, *KRAS* was significantly more prevalent in samples with lower expression levels of PSMC1 and PSMC2 (Figure 2D). In addition, PSMC family gene expression was significantly positively correlated with each other in LUAD (Figure 2E), supporting the hypothesis that PSMC genes should be studied collectively. The six PSMC family members were widely detected in LUAD samples from the HPA database (Figure 2F; Supplementary Figure S4), revealing their comprehensive existence in LUAD samples.

**FIGURE 3:**
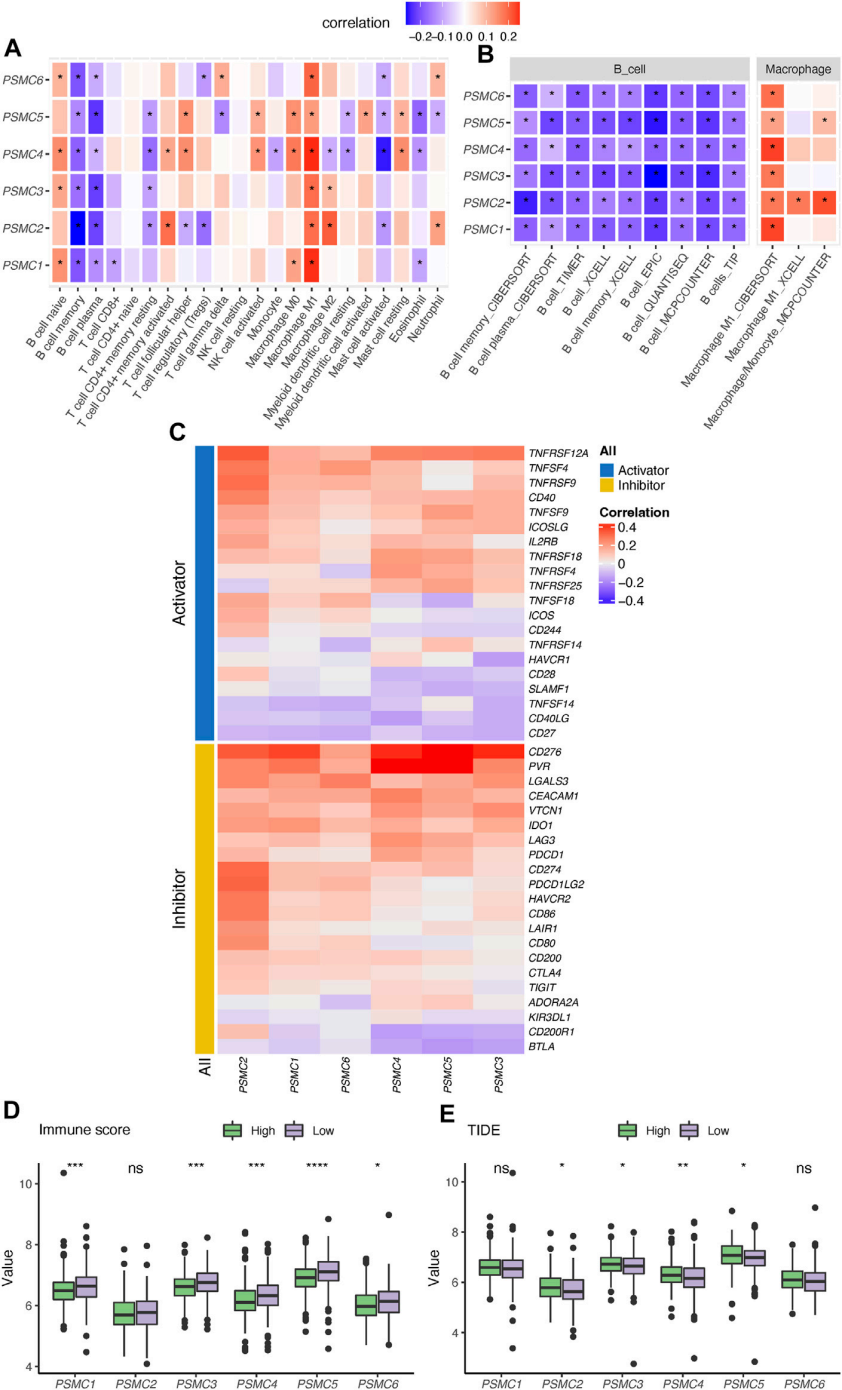
Correlation between PSMC genes and immune profile in lung adenocarcinoma (LUAD). **(A)** Correlation analysis between PSMC genes and the tumor-infiltrated immune cell profile in LUAD using the CIBERSOFT algorithm. **(B)** Correlation between the expression of PSMC genes and abundance of B cells and M1 macrophages revealed using multiple computational algorithms. **(C)** Correlation analysis between PSMC genes and immune checkpoints. Difference in the immune score **(D)** and T-cell dysfunction and exclusion (TIDE) **(E)** score between high- and low-expression groups. *p < .05, **p < .01, ***p < .001, and ****p < .0001.

Six PSMC genes exhibited different correlations with tumor-infiltrated immune cells but with concordance in the enrichment of M1 macrophages and exclusion of memory and plasma B cells revealed by the CIBERSOFT algorithm (Figure 3A). By performing integration analysis using multiple algorithms, we found that the exclusion of B cells and enrichment of M1 macrophages was a constant feature of all PSMC genes (Figure 3B). Notably, all PSMC genes were significantly positively correlated with immune checkpoint genes, including CD276 (B7-H3), PVR (CD155), and TNFRSF12A (Figure 3C). LUAD patients with PSMC gene overexpression had significantly lower immune scores, except for PSMC2 (Figure 3D; Supplementary Figure S3D). In contrast, PSMC2–4 genes were negatively correlated with the TIDE score, indicating an immune profile with dysfunction or exclusion in T cells (Figure 3E; Supplementary Figure S3E).

### 3.3 Identification of PSMC-related genes and construction of a PSMC family gene-based prognostic signature

**FIGURE 4:**
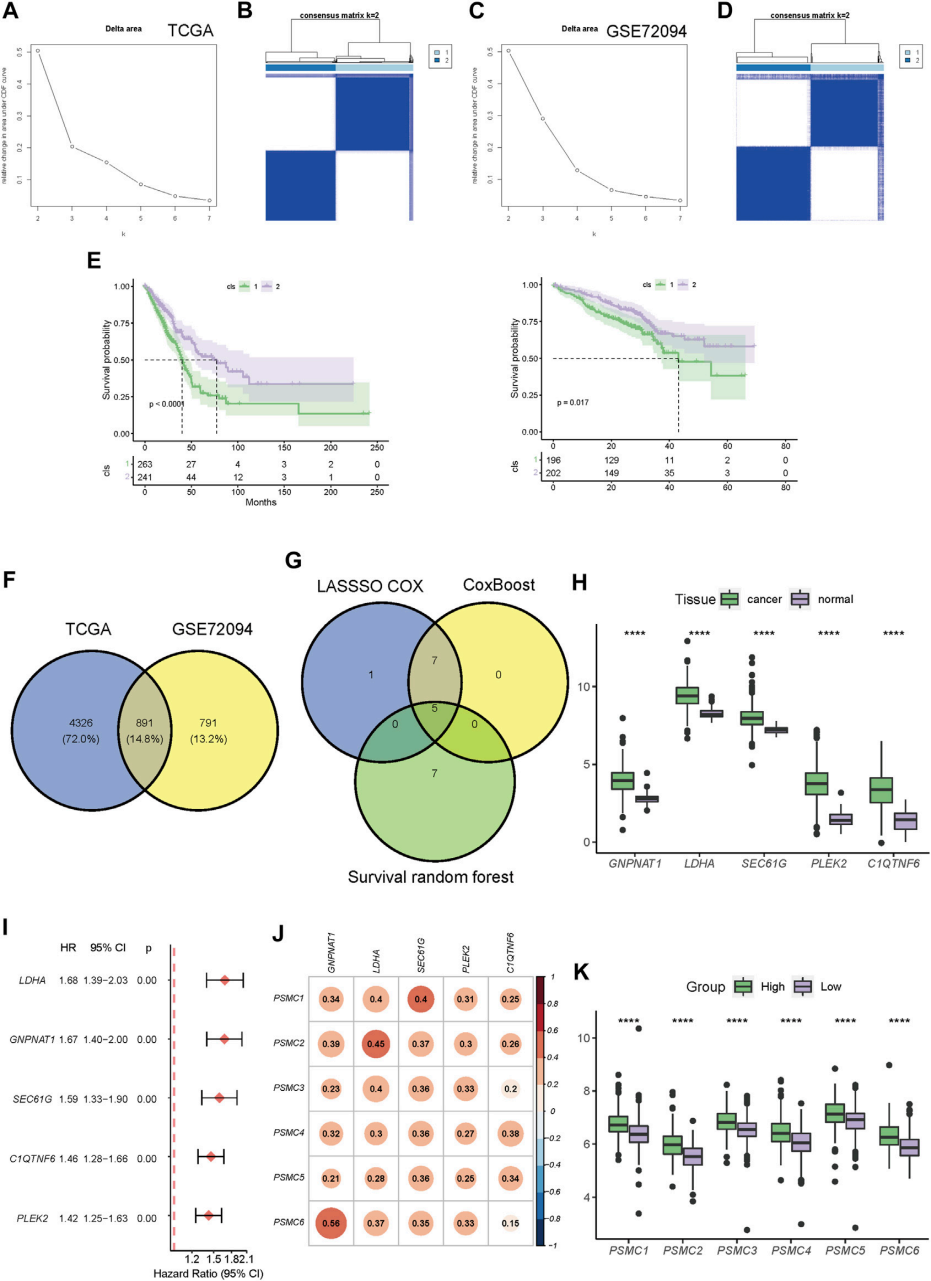
Construction of a PSMC gene-based prognostic signature. **(A)** CDF plot displaying the consensus distribution and the relative changes in the area under the CDF curve in the TCGA-LUAD cohort. **(B)** Consensus matrix heat map depicting consensus values on a white to blue color scale in TCGA-LUAD database. **(C)** CDF plot displaying the consensus distribution and the relative changes in the area under the CDF curve in the GSE72094 cohort. Y-axis, relative change in the area under the CDF curve and y-axis; k, the number of consensus clusters. **(D)** Consensus matrix heat map depicting consensus values on a white to blue color scale in the GSE72094 cohort. **(E)** Kaplan–Meier curves showing the overall survival of patients in different clusters in TCGA (left) and GSE72094 (right) cohorts. **(F)** Venn plot showing the overlapped cluster-related DEGs between the TCGA and GSE72094 cohorts. **(G)** Venn plot revealing the overlapped candidate genes found using LASSO-Cox, CoxBoost, and survival random forest. **(H)** Difference in the expression level of screened-out genes between tumor and normal tissues. **(I)** Forest plot of the hazard ratio (HR) of the candidate genes. **(J)** Pearson’s correlation coefficient between the expression levels of PSMC family genes and candidate genes. **(K)** Differences in the expression levels of PSMC family genes between high- and low-risk groups. Patients with LUAD in TCGA cohort were dichotomized according to the median risk score. *** *p* < .001.

Based on the expression pattern of PSMC family genes, unsupervised consensus clustering of LUAD samples in TCGA (Figures 4A, B) and GSE72094 datasets (Figures 4C, D) revealed two distinct clusters. It is noteworthy that patients from the two clusters had significantly different OS in both TCGA and GSE72094 cohorts (Figure 4E). A total of 5,217 and 1,682 DEGs were identified between the two clusters in TCGA and GSE72094 cohorts, respectively, of which 891 were shared by these two datasets (Figure 4F; gene lists are presented in Supplementary Table S3). DEGs in TCGA and GSE72094 cohorts were both significantly enriched in the cell cycle, proteasome, and DNA repair pathways, which revealed a consistent difference in the molecular function between PSMC clusters (Supplementary Figure S5). Then, through multiple machine learning algorithms including LASSO-Cox, CoxBoost, and survival random forest, five hub genes (GNPNAT1, LDHA, SEC61G, PLEK2, and C1QTNF6) were screened out (Figure 4G; details about candidate genes screened out by each machine learning algorithm are presented in Supplementary Table S4). All five genes were highly upregulated in tumor tissues relative to normal tissues (Figure 4H) and were associated with disease progression (Figure 4I). Meanwhile, the expression of all five genes was significantly positively correlated with the expression of the PSMC family genes (Figure 4J). The PSMC-related prognostic prediction signature was calculated using the following formula:

PSMC risk score = .101 × GNPNAT1 expression +.101 × LDHA expression +.0188 × SEC61G expression +.0739 × PLEK2 expression +.113 × C1QTNF6 expression.

Subsequently, LUAD patients in all training and validation cohorts were dichotomized into high- or low-risk groups according to the median risk score (Supplementary Figure S6). The high-risk group had significantly higher expression levels of the PSMC family genes than the low-risk group (Figure 4K).

### 3.4 Evaluation and validation of the PSMC signature

**FIGURE 5:**
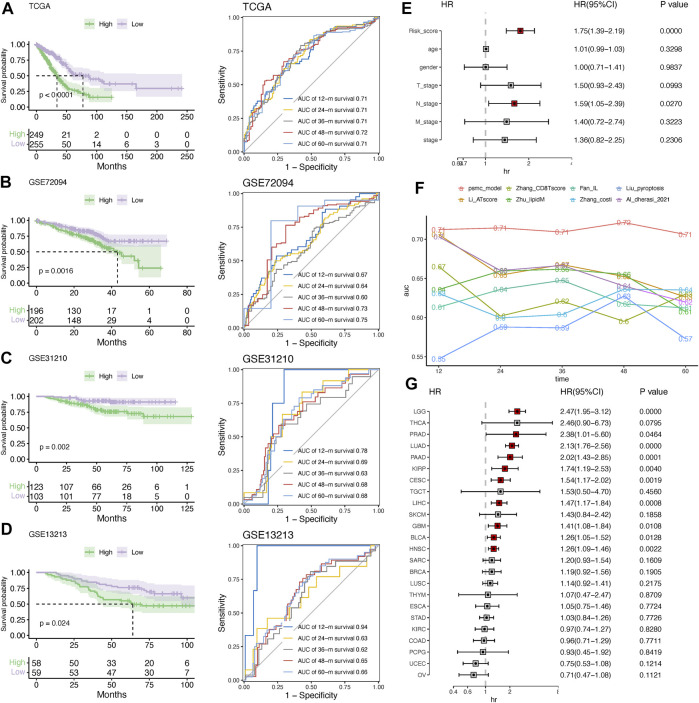
Evaluation and validation of the risk signature. Kaplan–Meier survival analysis and time-dependent ROC curves at 1–5 years in the training **(A)** and validation cohorts, including GSE72094 **(B)**, GSE31210 **(C)**, and GSE13213 **(D)**. **(E)** Multivariate Cox regression analysis of the hazard ratio (HR) of the PSMC risk score and other clinicopathological features in the training cohort. **(F)** Comparison of the predictive accuracy for 1- to 5-year survival between the PSMC signature and previously reported risk signatures in LUAD. **(G)** Forest plot showing the HR of the PSMC risk score from the TCGA dataset in pan-cancer.

To evaluate the predictive performance of the established PSMC signature, Kaplan–Meier survival and time-dependent ROC analyses were performed in both the training and validation cohorts. In the training cohort, the median OS (mOS) in the low-risk group was doubled compared to that in the high-risk group (77.27 vs. 34.20 months, *p* < .0001), and the prediction accuracy of the PSMC signature for survival at 1 to 5 years was >.71 (Figure 5A). Consistent with this result, low-risk patients in the validation cohorts (including GSE72094, GSE31210, and GSE13213) had significantly superior OS compared to high-risk patients (Figures 5B–D). In all three validation cohorts, the PSMC signature demonstrated robust and stable predictive accuracy for 1- to 5-year survival (Figures 5B–D). Multivariate Cox regression analysis revealed that the PSMC risk score was an independent risk factor in both the training (Figure 5E) and validation cohorts (Supplementary Figure S7). We then compared the predictive accuracy of the PSMC signature to that of previously reported LUAD risk signatures, and the results revealed that our risk signature outperformed these signatures in terms of survival prediction (Figure 5F). We also investigated the risk function of the PSMC signature in pan-cancer (Figure 5G) and found that, except for LUAD, a higher PSMC risk score indicated an inferior OS in the other nine cancer types, including low-grade glioma (LGG), prostate adenocarcinoma (PRAD), pancreatic adenocarcinoma (PAAD), kidney renal papillary cell carcinoma (KIRP), cervical squamous cell carcinoma, endocervical adenocarcinoma (CESC), liver hepatocellular carcinoma (LIHC), glioblastoma (GBM), urothelial bladder carcinoma (BLCA), and head and neck squamous cell carcinoma (HNSC).

### 3.5 Correlation of the signature with clinicopathological characteristics

**FIGURE 6:**
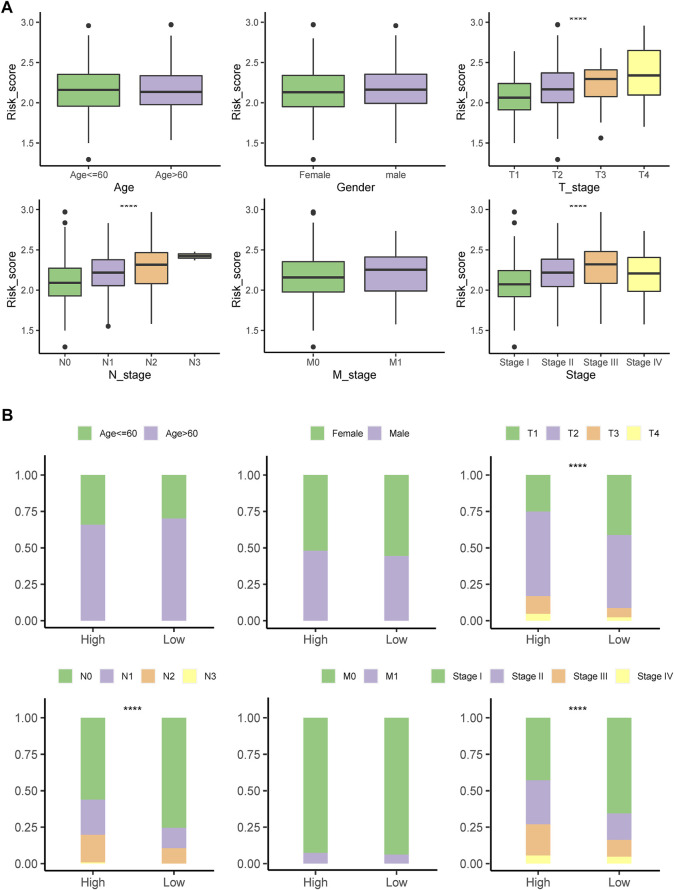
Correlation of the signature with clinicopathological characteristics. **(A)** Differences in the risk score between patients with different clinicopathological factors. **(B)** Comparing the distribution of patients with different clinicopathological factors between high- and low-risk groups. ****p < .0001; ns: not statistically significant.

Patients with LUAD at advanced tumor (T), lymph node (N), or neoplasm disease stage had significantly higher PSMC risk scores (Figure 6A). According to these findings, the high-risk group included more patients with advanced T, N, and tumor disease stages (Figure 6B).

### 3.6 Construction of a nomogram based on the risk score and clinical factors

**FIGURE 7:**
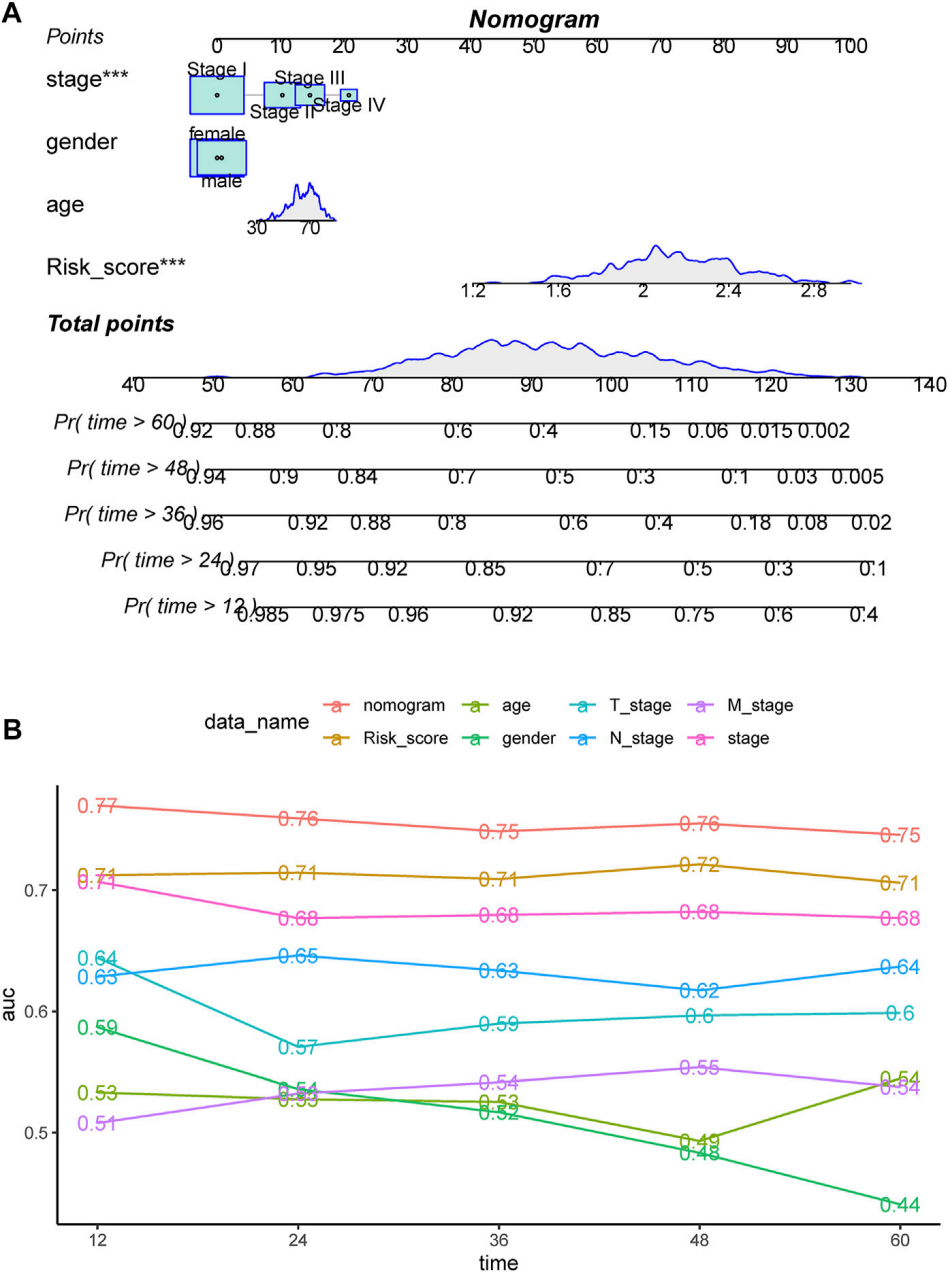
**(A)** Nomogram constructed for predicting probabilities of 1-, 3-, and 5-year overall survival (OS) in TCGA dataset by integrating the risk score and clinical factors, including stage, sex, and age. **(B)** ROC analyses of 1-, 3-, and 5-year OS for the nomogram and clinical factors.

By integrating the risk score with stage, sex, and age, a nomogram was developed that could robustly predict the survival probabilities at 1–5 years (Figure 7A). The AUC of the nomogram was >.75 at 1- to 5-year survival, outperforming the risk score alone and any other clinicopathological factors (Figure 7B).

### 3.7 Comprehensive analysis of the genomic profiles between high- and low-risk groups

**FIGURE 8:**
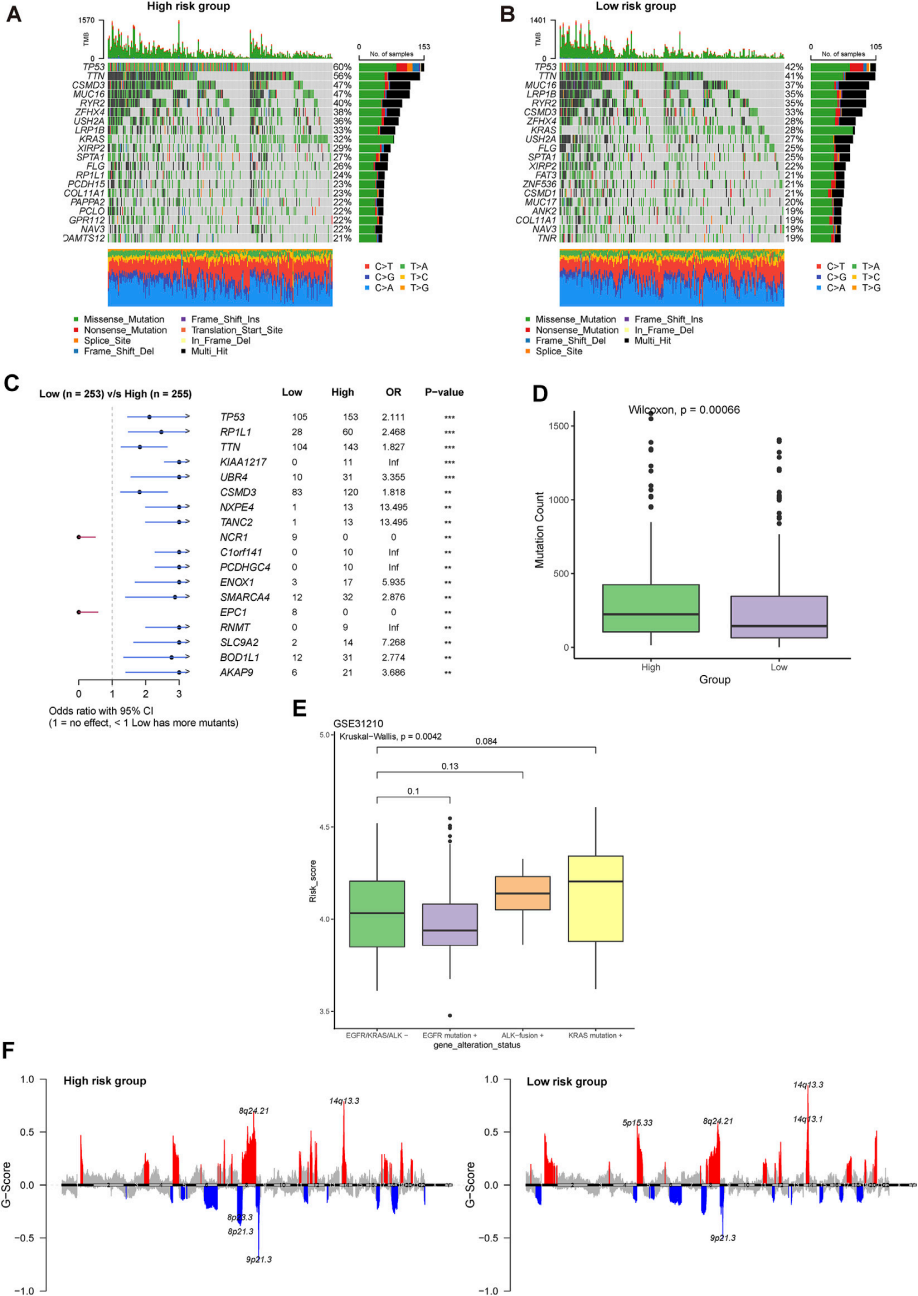
Comprehensive analysis of the genomic profiles between high- and low-risk groups. Oncoplots showing the top 20 prevalent genes in the **(A)** high and **(B)** low-risk groups. **(C)** Significantly different prevalent genes in the high- and low-risk groups. **p < .001 and ***p < .0001. **(D)** Differences in the mutation counts between different risk groups. **(E)** Differences in the risk group among LUAD patients with various driver genetic alterations in EGFR, KRAS, or ALK from the GSE31210 cohort. **(F)** Comparison of significant regions with amplification or deletion between the high- (left) and low- (right) risk groups.

The top altered genes in the high- and low-risk groups are shown in Figures 8A, B, respectively. Using Fisher’s exact test, the significantly different prevalence of the genes is illustrated in Figure 8C. Notably, almost all the significantly different genes were more prevalent in the high-risk group, including *TP53*, *RP1L1*, *TTN*, *CSMD3*, and *SMARCA4*, with the exception of *NCR1* and *EPC1*. Consistent with this feature, we found that the high-risk group had significantly higher mutation counts (Figure 8D). In the GSE31210 cohort, patients with LUAD with *EGFR* mutations had the lowest risk score, whereas those with *KRAS* mutations had the highest risk score (Figure 8E). Meanwhile, we found that more regions with CNV occurred in the high-risk group (Figure 8F). Consistent with the trend in genetic alterations, CNV was significantly more prevalent in the high-risk group, including representative amplification at 12p12.1, 12q15, 2q14.1, 3q26.2, 14q13.3, q24.21, and 8q21.13 and deletions at 17p13.3, 8p23.3, 9p21.3, and 8q24.21 (Supplementary Table S5). DEGs between the high- and low-risk groups were significantly enriched in the focal adhesion, extracellular matrix (ECM)-related pathway, cell cycle, and p53 pathway (Supplementary Figure S8).

### 3.8 PSMC signature was correlated with the immune profile in LUAD

**FIGURE 9:**
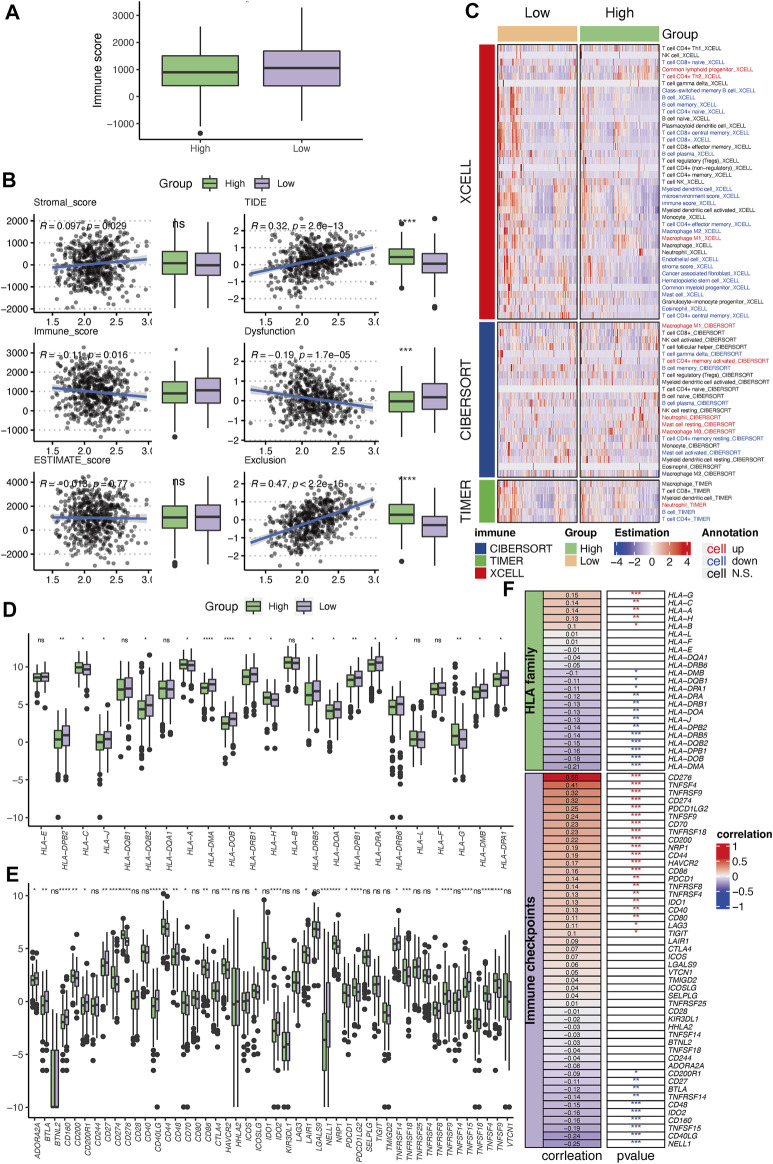
Immune profile correlating with the established PSMC gene-based prognostic signature. **(A)** Differences in the immune, stromal, and ESTIMATE scores between the high- and low-risk groups. **(B)** Correlation between PSMC risk and TIDE scores. **(C)** Tumor-infiltrated lymphocyte profile analyzed using XCELL, CIBERSOFT, and TIMER. **(D)** Differences in the expression levels of HLA family genes between the high- and low-risk groups. **(E)** Differences in the expression levels of immune checkpoint genes between the high- and low-risk groups. **(F)** Correlation between the PSMC risk score and the expression of HLA family genes or immune checkpoints. *p < .05, **p < .01, ***p < .001, and ****p < .0001; ns: not statistically significant.

The high-risk group had a significantly lower immune score than that of the low-risk group (Figure 9A). The PSMC risk score was significantly positively correlated with the TIDE score (R = .32), which was mostly attributable to the T-cell exclusion feature (R = .47, Figure 9B). The abundance of tumor-infiltrated lymphocytes was investigated using XCELL, CIBERSOFT, and TIMER (Figure 9C). Combined with the results from these three algorithms, the high-risk group had a lower immune-inflamed TME characterized by a higher abundance of M1 macrophages but a lower abundance of CD4^+^ T cells and B cells, which was consistent with the aforementioned findings in the TME related to PSMC family genes. Moreover, more HLA family genes were upregulated in the low-risk group, showing a negative correlation between the PSMC risk score and HLA family genes (Figures 9D, F). In contrast, a positive correlation was identified between the risk score and HLA-G, HLA-C, HLA-A, and HLA-B (Figures 9D, F). Regarding the association between immune checkpoints, we found 21 immune checkpoints that were significantly positively correlated with the PSMC risk score, particularly CD276, TNFSF4, TNFRSF9, and CD274 (Figures 9E, F). Meanwhile, NELL1, CD40LG, and eight other immune checkpoints were negatively correlated with the PSMC risk score.

### 3.9 Evaluation of the therapeutic response to ICI

**FIGURE 10:**
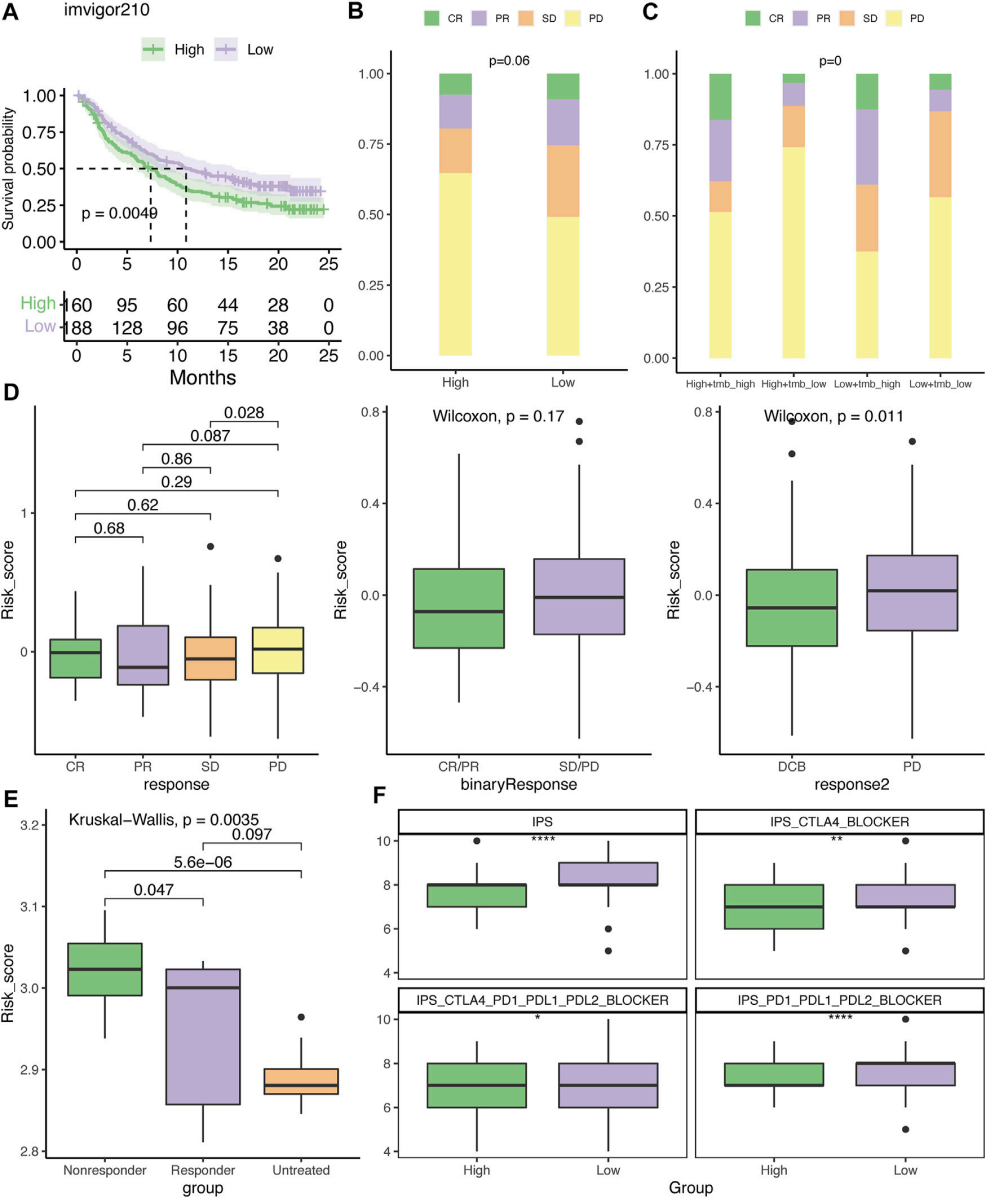
Evaluation of the therapeutic response to the immune checkpoint inhibitor. **(A)** Kaplan–Meier plot of overall survival for patients with low- or high-risk scores in the IMvigor210 cohort. The distribution of patients who had CR, PR, SD, or PD to atezolizumab in the high- and low-risk groups **(B)** or with TMB stratification **(C)**. **(D)** Differences in the risk score among patients who had different clinical benefits from atezolizumab. **(E)** Difference in the risk score among mice who had different clinical benefits to CTLA4 blockades in the GSE63557 dataset. **(F)** Association between IPS and risk score. DCB, durable clinical benefit; CR, complete response; PR, partial response; SD, stable disease; and PD, progressive disease. *p < .05, **p < .01, and ****p < .0001.

Based on the difference in the immune profiles between the high- and low-risk groups, we hypothesized that low-risk patients may be more likely to respond to ICIs. In the IMvigor210 cohort, patients in the low-risk group had better survival rates than those in the high-risk group (Figure 10A). Relatively more patients with an objective response (CR/PR) or SD were present in the low-risk group (Figure 10B). By integrating with the TMB value, it was demonstrated that patients in the high-TMB/low-risk group had more patients with DCB to the ICI (Figure 10C). Furthermore, patients with PR and PD had the lowest and highest risk scores, respectively (Figure 10D). Although there was no significant difference between patients with and without the objective response to atezolizumab, patients with DCB to this ICI regimen had notably lower risk scores than those with PD (Figure 10D). In concordance with this result, responders to the CTLA4 blockade in the GSE63557 cohort also had a significantly reduced risk score (Figure 10E). In addition, the IPS, IPS-CTLA4, IPS-CTLA4-PD1-PD-L1-PD-L2, and IPS-PD1-PDL1-PDL2 scores were significantly higher in the low-risk group (Figure 10F).

### 3.10 Evaluation of chemotherapy response

**FIGURE 11:**
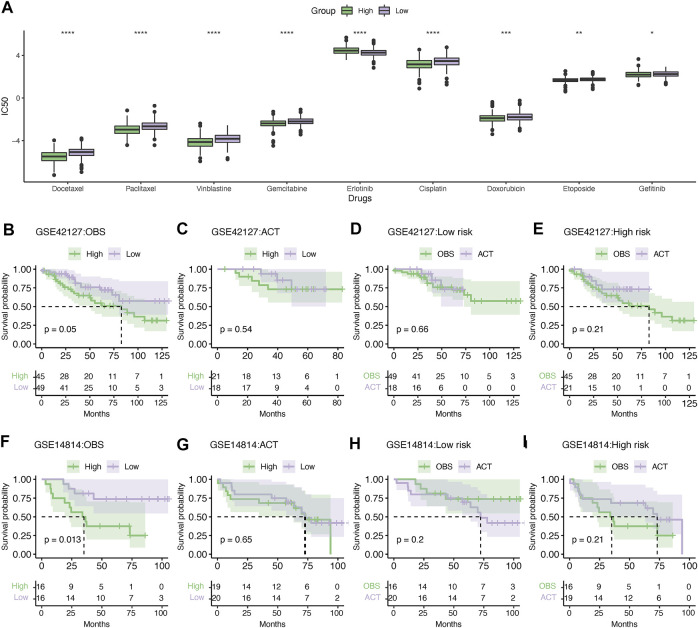
Evaluation of chemotherapy response. **(A)** Differences in the sensitivity of chemotherapy between high- and low-risk patients. *p < .05, **p < .01, ***p < .001, and ****p < .0001. Kaplan–Meier survival analysis of the LUAD patients administered without **(B)** or with **(C)** adjuvant chemotherapy in the GSE42127 dataset. Kaplan–Meier survival analysis showing the survival difference in the low- **(D)** and high-risk groups **(E)** treated with or without ACT in the GSE42127 dataset. Similarly, Kaplan–Meier survival analysis showing the difference in survival between the high- and low-risk patients without **(F)** or with ACT **(G)** in the GSE14814 dataset. Kaplan–Meier survival analysis of low- **(H)** or high-risk patients **(I)** treated with ACT or observation alone. IC50, the half maximal inhibitory concentration; ACT, adjuvant chemotherapy; and OBS, observation.

Based on the analysis of the GDSC database, it was demonstrated that the high-risk group was more chemosensitive, with lower IC50 values for docetaxel, paclitaxel, vinblastine, gemcitabine, cisplatin, doxorubicin, and etoposide (Figure 11A). However, the low-risk group was significantly more sensitive to erlotinib than the high-risk group. Next, we compared the clinical benefits between high- and low-risk LUAD patients treated with adjuvant chemotherapy (ACT) in the GSE42127 and GSE14814 datasets. In the absence of ACT, high-risk patients had significantly shorter survival than low-risk patients in the GSE42127 dataset (Figure 11B). However, this difference diminished when ACT was administered (Figure 11C). It is noteworthy that ACT did not improve the outcomes for low-risk patients (Figure 11D); in contrast, high-risk patients treated with ACT had a relatively longer survival than those treated with observation alone; however, the difference was not statistically significant due to the limitation in the sample size (Figure 10E). The GSE14814 dataset corroborated these findings (Figures 11F–I).

### 3.11 External validation

**FIGURE 12:**
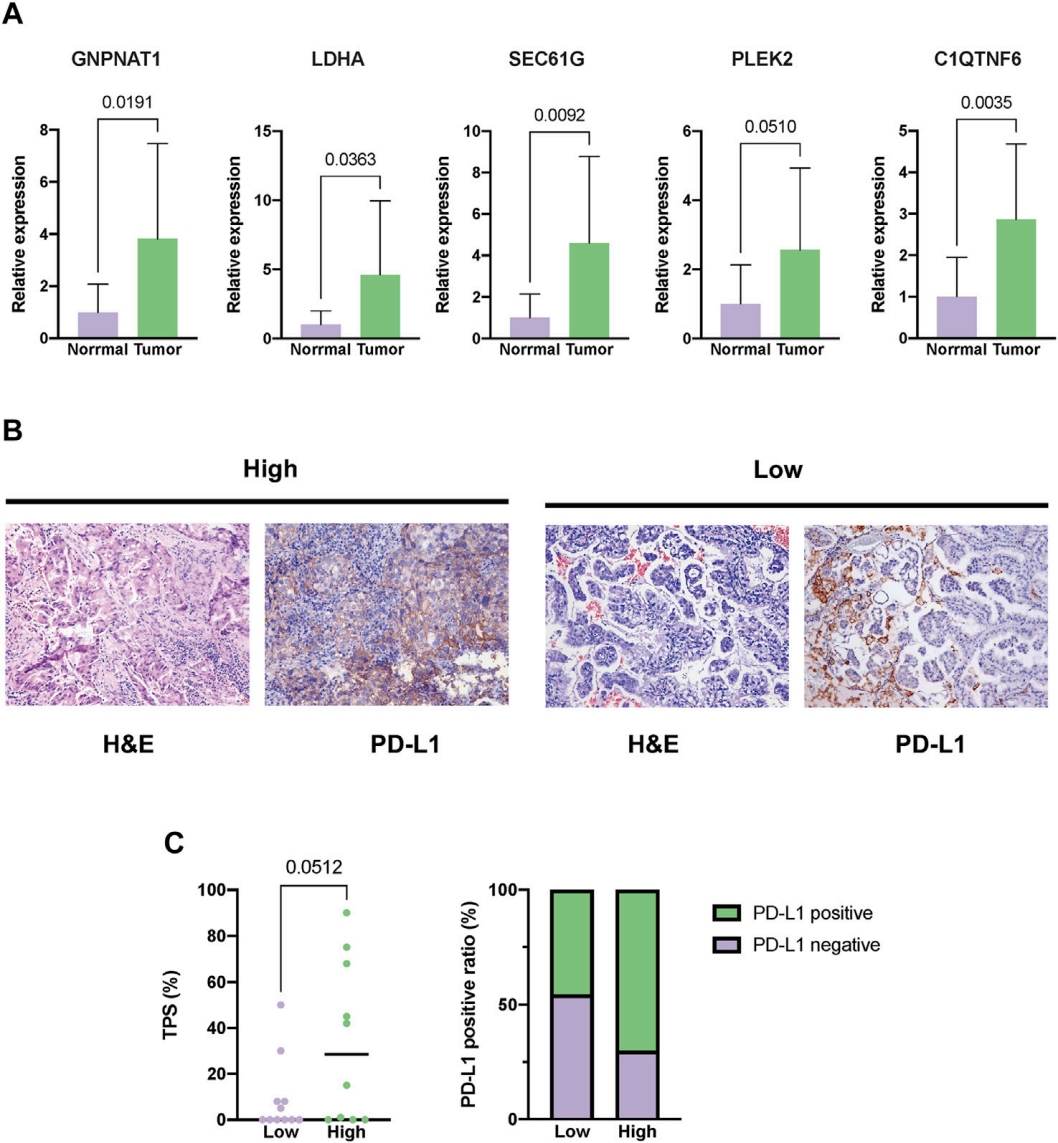
External validation in our local cohorts. **(A)** Evaluating the expression difference between tumor (*n* = 21) and normal tissues (*n* = 11) using real-time polymerase chain reaction (PCR). **(B)** Representative images of PD-L1 staining of tumor tissues from the high- or low-risk LUAD patients. **(C)** Difference in the expression level between the high- (*n* = 10) and low-risk groups (*n* = 11). The expression level of PD-L1 was depicted as the tumor proportion score (TPS).

First, in our local cohorts, we compared the expression levels of the five hub genes in tumor and normal tissues and found that all five genes were overexpressed in tumor tissues (Figure 12A). The 21 LUAD patients were also dichotomized into the high- (n = 10) and low-risk (n = 11) groups based on the median risk score calculated using the same formula. As we identified a positive correlation between the risk score and PD-L1 expression in TCGA cohort, we also validated the variance in PD-L1 expression levels in the local cohort. A higher TPS level and PD-L1-positive fraction were identified in the high-risk group (Figures 12B, C).

## 4 Discussion

Although a number of prior studies have suggested that all the PSMC genes were oncogenic in a single tumor type, we revealed that LUAD was the only cancer type in which all six PSMC genes were associated with a worse outcome by integrated pan-cancer analysis. Through immunochemistry analysis of the HPA database, all six PSMC genes were found to be differentially expressed in the LUAD samples, validating their expression and prognostic significance (Wang et al., 2020; Kao et al., 2021). A single *in vitro* investigation revealed that PSMC6 suppression could reduce the growth and metastasis of LUAD cells, leaving the function of additional PSMC genes in LUAD unknown (Zhang et al., 2021). These unfavorable prognostic functions were consistently associated with a distinct genomic and immune profile in LUAD, including increased TMB and CD276 expression, an immune cold feature characterized by a lower immune score due to the exclusion of B cells, enrichment of M1 macrophages, and a higher TIDE score. This feature was also shared by the PSMC risk signature that was established based on the expression pattern of PSMC family genes in LUAD. In addition, proteasome inhibitors, such as bortezomib, carfilzomib, and ixazomib, are effective antitumor drugs for treating multiple myeloma (Kumar et al., 2014). The expression levels of PSMC family genes are related to the sensitivity to proteasome inhibitors (Acosta-Alvear et al., 2015), which have been widely investigated in lung cancer (Rohondia et al., 2020). We also found that the upregulation of PSMC family genes was correlated with immune regulation-related pathways, DNA replication, endocytosis, and cell cycle pathways. Consequently, it is possible that the overexpression of PSMC family genes is a result of an increase in tumor immunology and cell metabolism, which is also supported by the results from previous studies (Qin et al., 2019; Duan et al., 2021b; He et al., 2021; Kao et al., 2021; Yang et al., 2021).

Five genes have been identified as regulators or effectors that are involved in PSMC-related disease progression in LUAD. Metabolic reprogramming is a hallmark of malignancy that satisfies the growing need for the active metabolism and proliferation of cancer cells. The hexosamine biosynthetic pathway (HBP) is a key glucose- and nitrogen-related metabolism pathway in which glucosamine-phosphate N-acetyltransferase 1 (GNPNAT1) plays an essential regulatory function (Kaushik et al., 2016). However, little is known regarding the specific functions of GNPNAT1 and HBP in lung cancer. Kim et al. discovered that hindering HBP through the inhibition of key enzymes involved has antitumor effects preferentially in lung cancer with a KRAS/LKB1 co-mutant (Kim et al., 2020). In line with the findings of the present study, another study also found that upregulation of GNPNAT1 was associated with an inferior outcome in LUAD, combined with a negative correlation between B cells and CD4^+^ T cells (Liu W. et al., 2021). Consistent with our results, other PSMC signature-related genes, including LDHA (Wu et al., 2020), SEC61G (Zheng et al., 2021), PLEK2 (Wu et al., 2020), and C1QTNF6 (Zhang & Feng, 2021), have been shown to be risk genes in lung cancer *in vitro*, mediating the development, growth, and metastasis of lung cancer. However, none of them has previously uncovered a direct or indirect relationship with PSMC or proteolysis, with the exception of C1QTNF6, which is involved in the complement activation pathway and participates in the proteolytic cascade (Murayama et al., 2015). As a pivotal constituent of the TME, the complement cascade is involved in the regulation of the innate immune system, and previous studies have indicated that the complement activation alternative pathway might play a crucial dual role in carcinogenesis (Su et al., 2020). As cancer cells could benefit from the immunosuppressive effect caused by complement activation, therapeutic strategies targeting the complement pathway have been suggested as potential therapies for patients with LUAD that could be developed in the future (Kleczko et al., 2019). To the best of our knowledge, this is the first study to elucidate their significance in the prognostic regulatory process of PSMC family genes in LUAD; however, additional research is required to offer deeper insights into the regulatory network between PSMC and these genes.

The PSMC signature that we established had a robust and stable performance in distinguishing LUAD patients with worse outcomes. Although it was primarily explored in LUAD, pan-cancer analysis revealed that it also has predictive value for numerous cancer types, thus validating its importance. The genomic alterations and CNV associated with the risk score may contribute to inferior survival in high-risk patients. High-risk patients had a significantly higher prevalence of TP53 and SMARCA4 alterations, which were both demonstrated to be risk biomarkers associated with worse survival in lung cancer patients (Jiao et al., 2018; Schoenfeld et al., 2020). Consistent with genomic alterations, CNV features were also attributed to the difference in survival between the high- and low-risk groups. More Amp12p12.1 (KRAS), Del17p13.3 (TP53), and Amp12q15 (CDK4 and MDM2) mutations have been found in the high-risk group, and genes involved in these focal regions are not only associated with prognosis (Wagner et al., 2011) but also with treatment response in LUAD (Kato et al., 2017; Sitthideatphaiboon et al., 2022). This significant genomic and chromosomal instability associated with the PSMC signature may be caused by dysfunction in the DNA repair pathways (Bakhoum and Cantley, 2018), which are the main signaling pathways correlated with PSMC gene expression. Numerous studies have demonstrated that the TME plays a crucial role in tumorigenesis, progression, and metastasis of LUAD (Chen and Zhou, 2021; Wei et al., 2021). Another study found that the survival of patients with LUAD was associated with tumor-infiltrating immune cell levels in the TME (Jin et al., 2021).

Great advances have been made in treating patients with LUAD, especially with the rapid development of immunotherapy, and patient survival has significantly improved (Sun et al., 2020; Wen et al., 2021). However, only a quarter of LUAD patients respond to PD-1/PD-L1 inhibitor monotherapy (Reck et al., 2022). TMB is an important predictor of response to immunotherapy, and higher TMB has been shown to be positively correlated with better immunotherapy response in various types of tumors (Yarchoan et al., 2017). Although we found that LUAD patients in the high-risk group had significantly higher TMB levels, it was suggested that they were less sensitive to ICIs. Despite wide acknowledgement that a high TMB value (>10 mutations/MB) is associated with elevated neoantigen load, which could be recognized by the immune system and thus increase the sensitivity of immunotherapy (McGranahan et al., 2016), increasing contradictory evidence has emerged challenging the application of TMB as a reliable biomarker for ICI (McGrail et al., 2021). As the cancer-immunity cycle comprises seven steps, of which the release of cancer cell antigens is the first, malfunctions in other steps may also hinder the efficacy of ICIs (Chen Daniel and Mellman, 2013). We found a notable downregulation of HLA family members in high-risk groups, indicating a relative defect in cancer antigen presentation or T-cell priming and activation (Braun et al., 2021). Meanwhile, the TME may also contribute to a lower response to ICIs in the high-risk group, especially if there is a deficiency in CD8^+^ T cells and B cells. A previous study found that the level of peripheral IgM+ memory B cells could serve as a positive biomarker for predicting the efficacy of PD-1 monotherapy in NSCLC patients (Xia et al., 2021). Meanwhile, the lack of tumor-infiltrated CD8^+^ T cells has been suggested as a reason for primary resistance to immune checkpoint inhibitors (Horton et al., 2021). Interestingly, the negative correlation between the PSMC signature and TILs may be a reason for the lower immune score and possible lower efficacy of immunotherapy for high-risk patients even with high TMB values and neoantigen loads. In this study, multiple immune checkpoints were positively correlated with the risk score, especially CD276 and CD274. CD276, also known as B7 homolog 3 protein (B7-H3), is overexpressed in tumor cells and contributes to the development of lung cancer (Yonesaka et al., 2018). The combination of blocking CD276 and PD-1 using antibodies successfully inhibited tumor growth and increased tumor-infiltrated CD8^+^ T and NK cell levels (Lee et al., 2017). Notably, blocking CD276 using multiple strategies, such as antibody–drug conjugates, antibody-dependent cell-mediated cytotoxicity, antibodies, and CAR-T cells, have been evaluated in clinical trials and have shown promising antitumor effects (Zhou and Jin, 2021).

There are several limitations to this study. Due to the lack of coexistence of genomic and transcriptomic data in other validation datasets, the genomic feature correlated with PSMC genes or the signature was investigated only in TCGA cohort. Even though the current study is the first to our knowledge to identify the unique genomic feature associated with PSMC genes or its signature, additional research is required to confirm the association. Second, based on the genomic, TME feature, and immunotherapy dataset enrollment, we suggested the application of PSMC signature in predicting the responders to immunotherapy. Unfortunately, we were unable to access any LUAD cohort with both transcriptome and treatment responses to immunotherapy to conduct bioinformatics analyses. Also, in the meantime, TCGA cohort did not collect details of treatment information, therefore prohibiting us from analyzing the relationship between treatment selection and patient survival stratified by the risk score. Future prospective studies should be conducted to confirm our findings using large cohorts with additional long-term survivors. In addition, although we conducted some validation tests using local LUAD samples, the sample size was small. Also, because the RNA sample we collected was insufficient for RNA-seq, many of the public datasets’ findings were not confirmed in our local cohorts. Because we did not perform mechanistic analysis, the regulatory pathway was not elucidated in detail. To investigate it, additional experimental methods are required.

Thus, we estimated the expression level of PSMC gene family members and constructed a prognostic signature based on the integrative expression pattern of PSMC gene family members. Furthermore, the correlation between clinical characteristics, TME, patient prognosis, and risk score was identified. Significant differences were observed in the percentage of immune cells and the prognosis of patients between the low- and high-risk groups. In addition, there was a statistically significant difference in the response to immunotherapy and chemotherapy between the low- and high-risk groups. Our results indicate that PSMC gene family members play a pivotal role in predicting the prognosis of LUAD patients and provide guidelines for oncologists to select optimal strategies such as immunotherapy, chemotherapy, and combination therapy.

## Data Availability

The original contributions presented in the study are included in the article/Supplementary Material; further inquiries can be directed to the corresponding authors.

## Supplementary material

Supplementary Figure S1 Schematic diagram of the study design.

Supplementary Figure S2
**(A)** Oncoprint of PSMC gene alterations in TCGA Pan-Cancer Atlas Study. **(B)** Oncoprint showing genomic alterations of PSMC genes in TCGA-LUAD dataset. **(C)** Heatmap showing clustering of the CpG methylation levels of PSMC genes. **(D)** Correlation analysis between the CpG methylation level and mRNA expression level of each PSMC gene. **(E)** Association between the CpG methylation level of each PSMC gene and overall survival in LUAD.

Supplementary Figure S3
**(A)** Correlation between each PSMC gene and the clinical feature, including sex, tumor (T), lymph node (N), long-distance metastasis stage (M), and neoplasm disease stage in LUAD. **(B)** Difference in the mRNA expression level of each PSMC gene among normal and tumor tissues with different clinical stages. Correlation analysis between the expression of PSMC genes and mutation count **(C)**, immune score **(D)**, and TIDE score **(E)**. LUAD, lung adenocarcinoma.

Supplementary Figure S4 Expression pattern of the protein level of each PSMC gene in the HPA database.

Supplementary Figure S5 Pathway enrichment of PSMC cluster-related differentially expressed genes (DEGs) in TCGA and GSE72094 cohorts.

Supplementary Figure S6 Distribution of high- and low-risk patients in the TCGA, GSE72094, GSE31210, and GSE13213 cohorts.

Supplementary Figure S7 Multivariate Cox regression analysis of the hazard ratio (HR) of the PSMC risk score and other clinicopathological features in the GSE72094 and GSE31210 cohorts.

Supplementary Figure S8 Pathway enrichment of differentially expressed genes (DEGs) between high- and low-risk groups in TCGA cohort.
