# Supplementary figures and images for: Pan-cancer analysis identifies proteasome 26S subunit, ATPase (PSMC) family genes, and related signatures associated with prognosis, immune profile, and therapeutic response in lung adenocarcinoma
Source: Front Genet. 2023 Jan 9;13:1017866. doi: 10.3389/fgene.2022.1017866 (PMC9868736; doi:10.3389/fgene.2022.1017866)

A

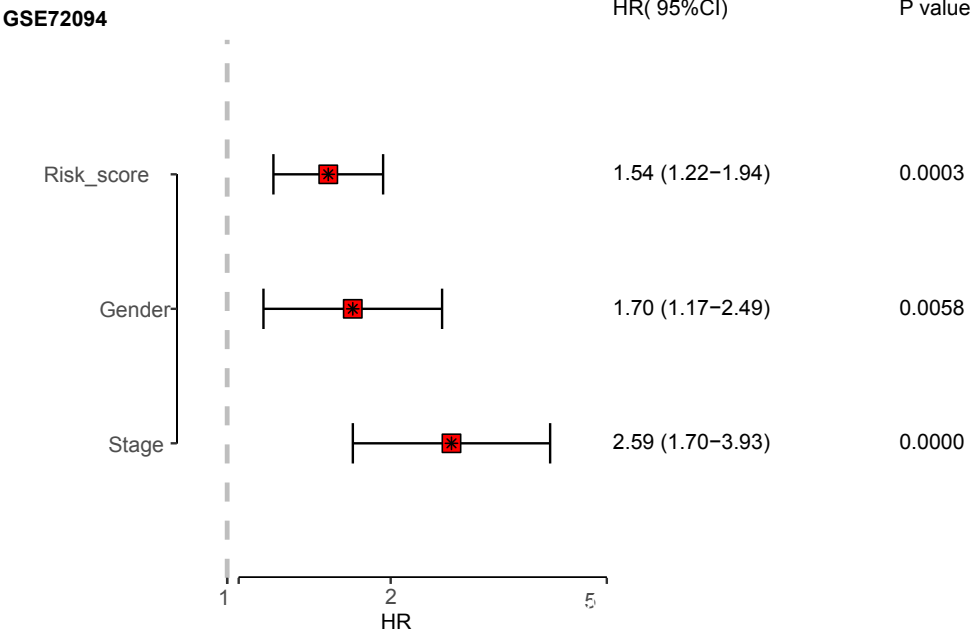

B

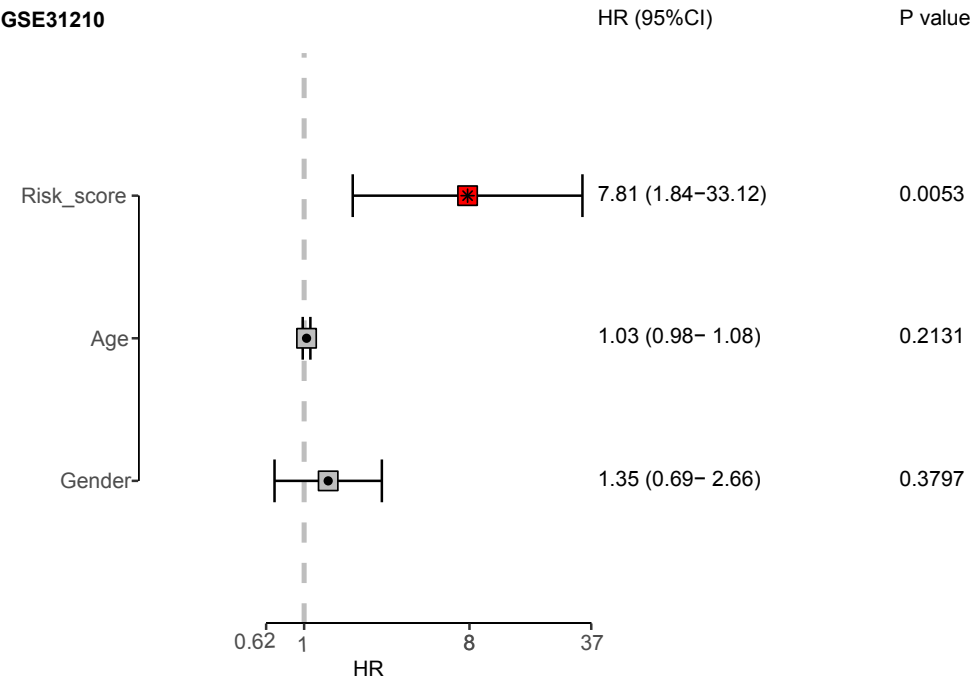

Supplement: Supplementary file 1 [file DataSheet7.PDF]

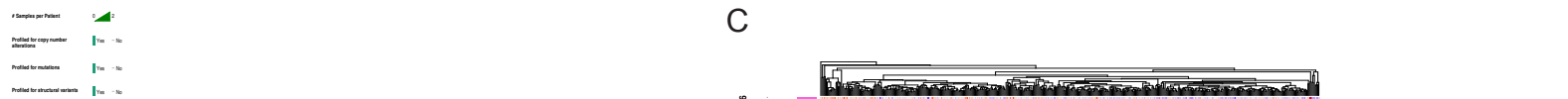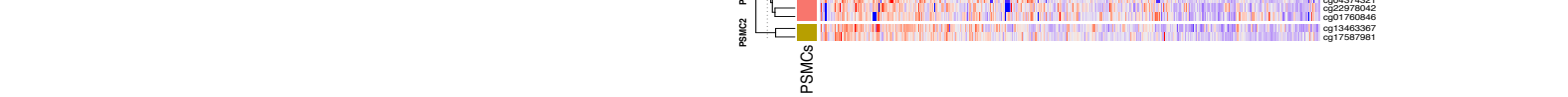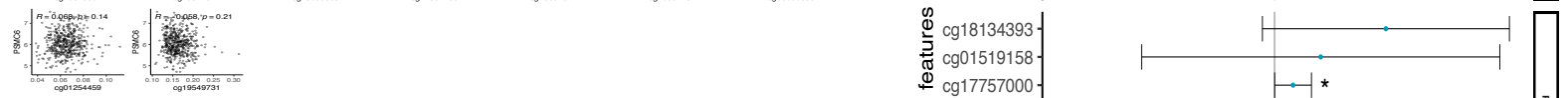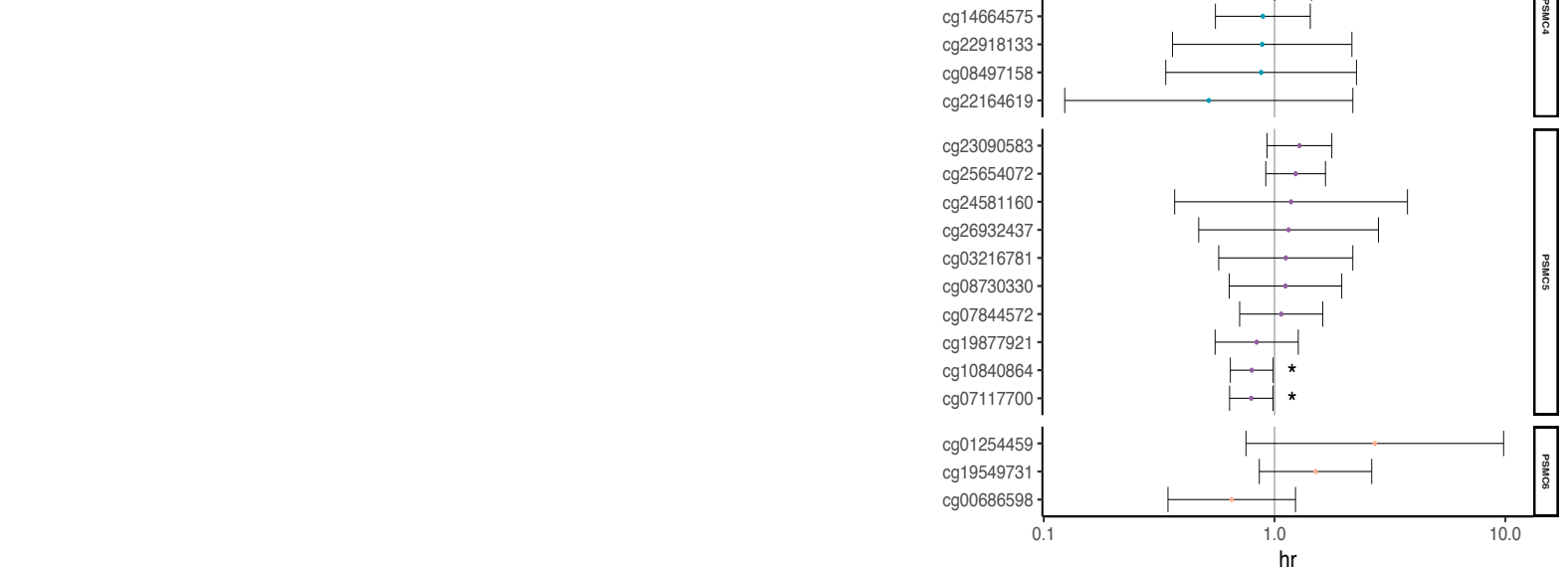

Supplement: Supplementary file 2 [file DataSheet2.PDF]

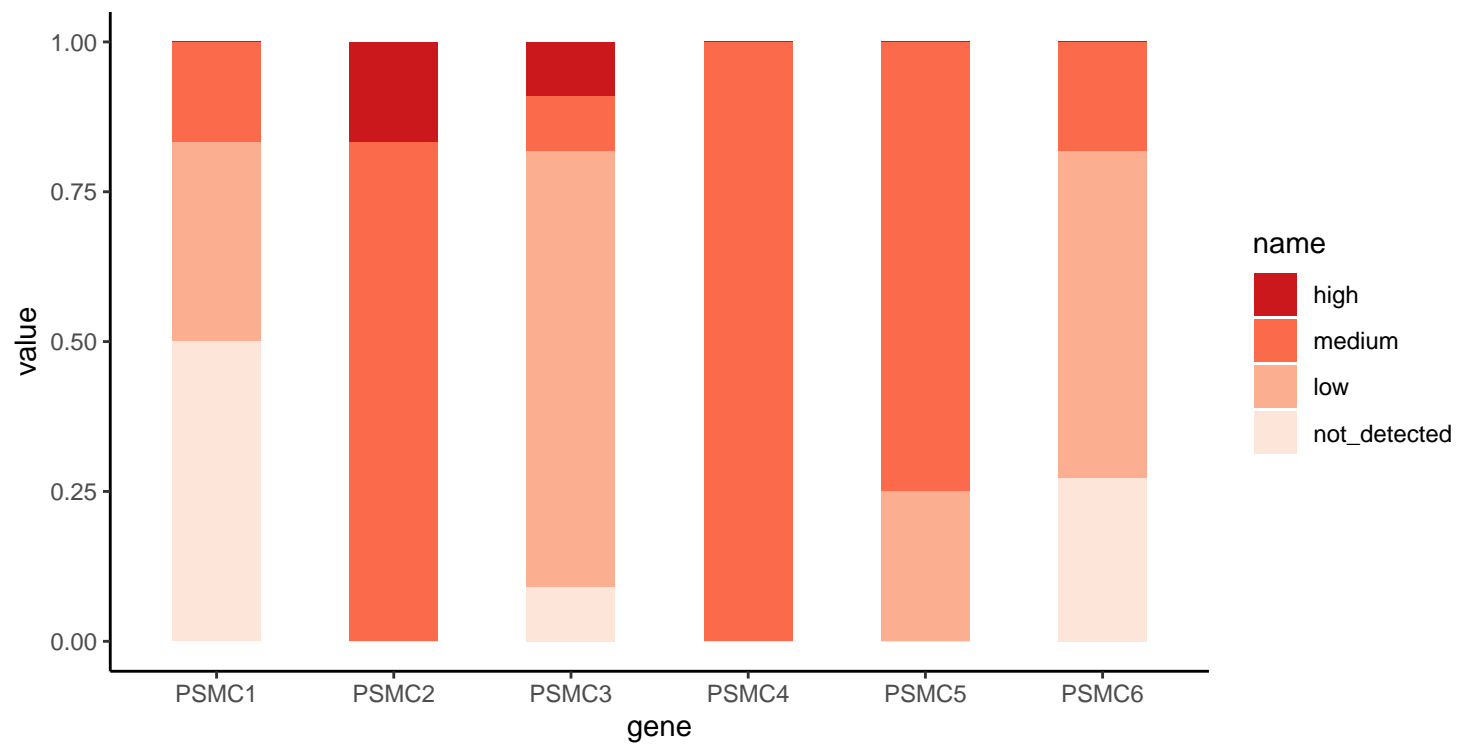

Supplement: Supplementary file 4 [file DataSheet4.PDF]

tcga

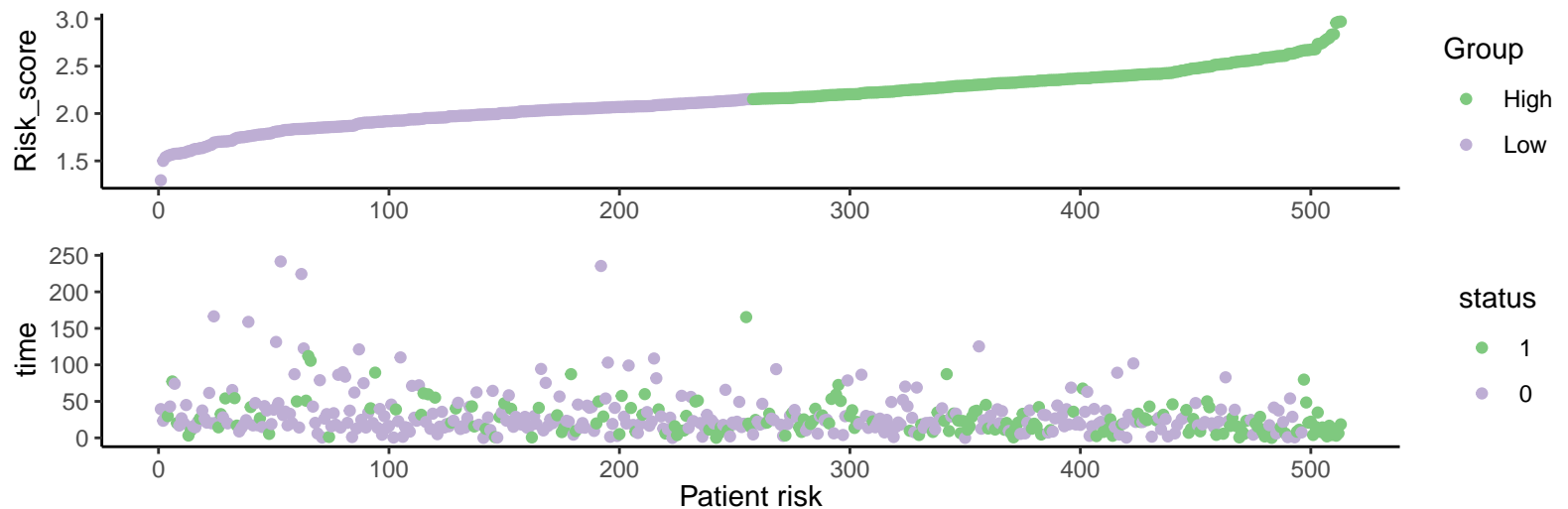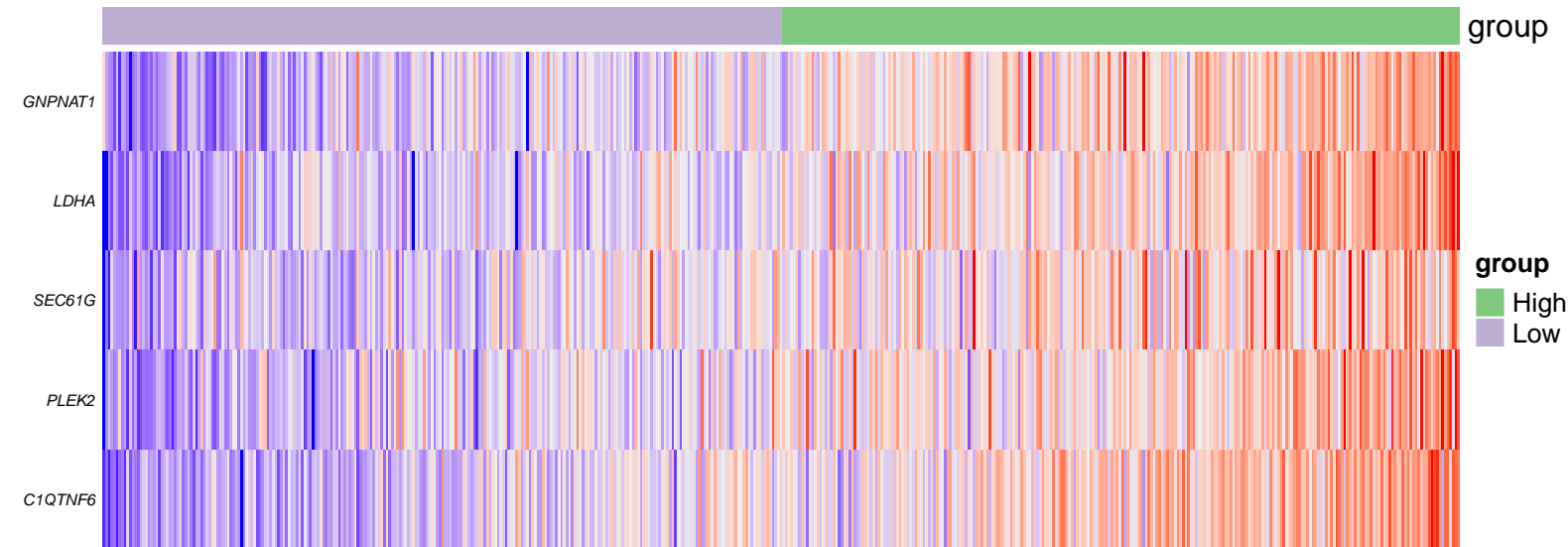

gse72094

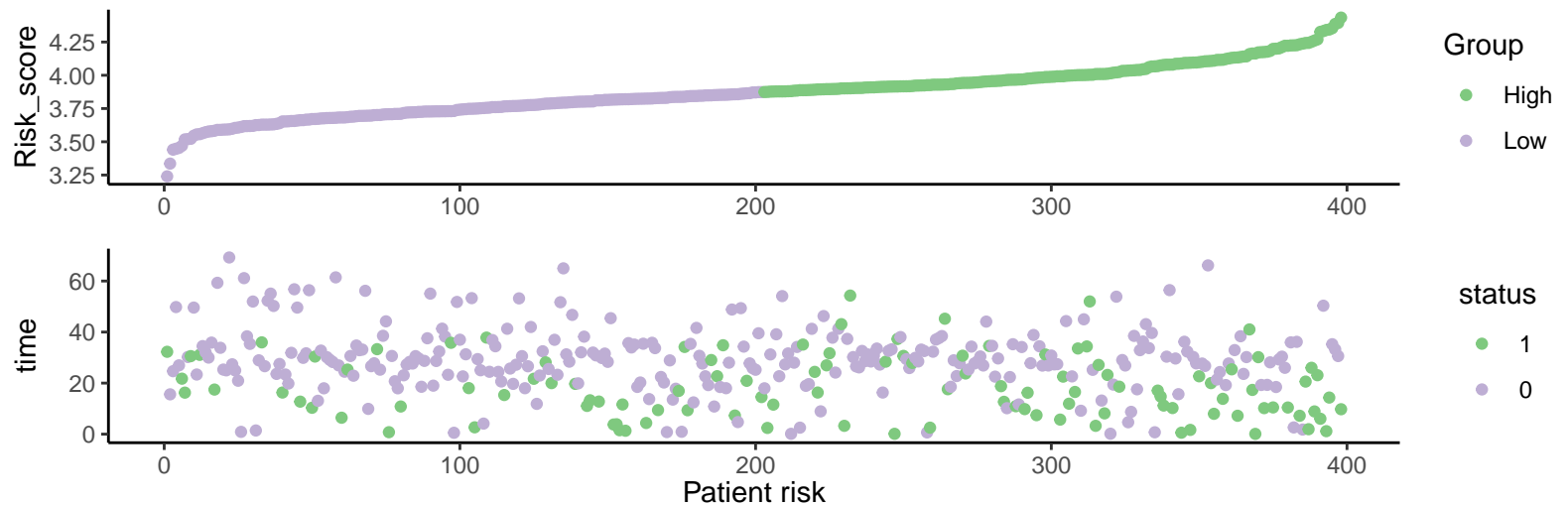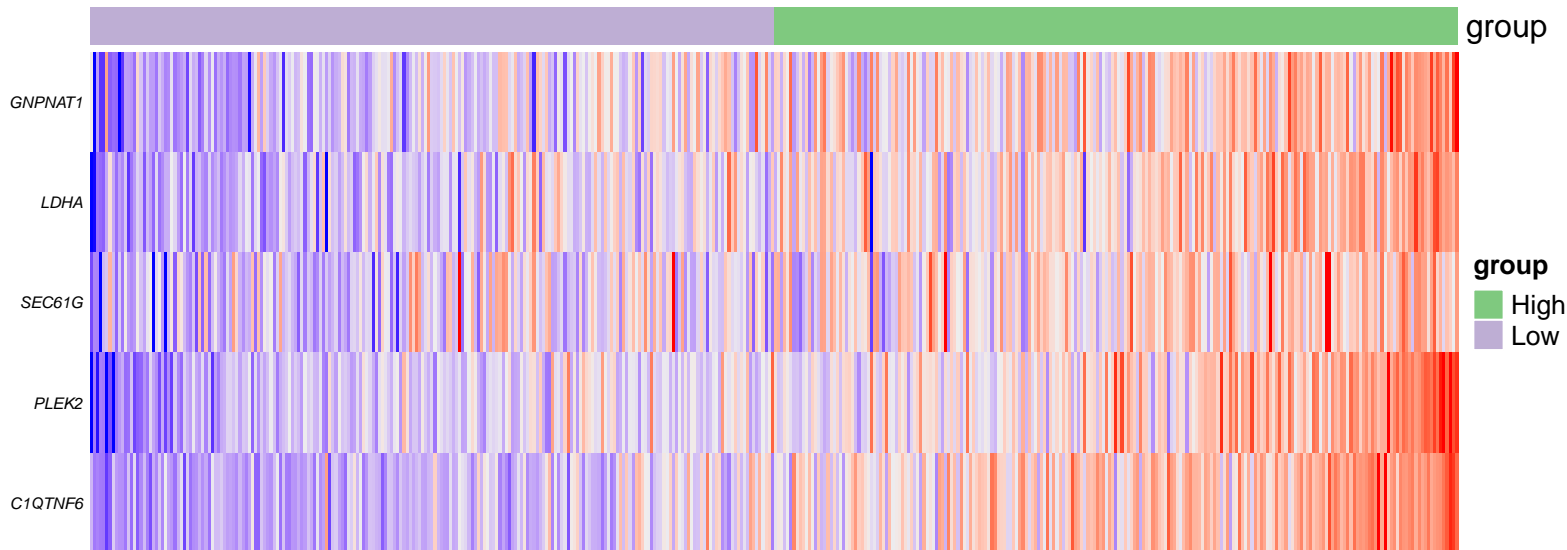

gse31210

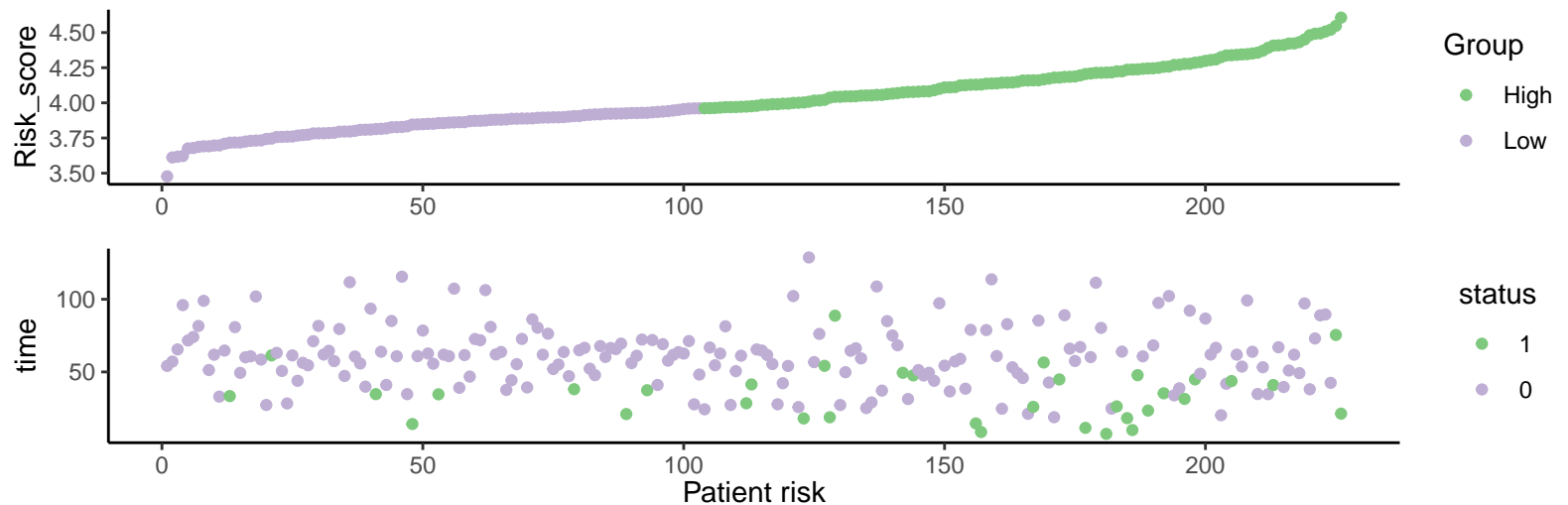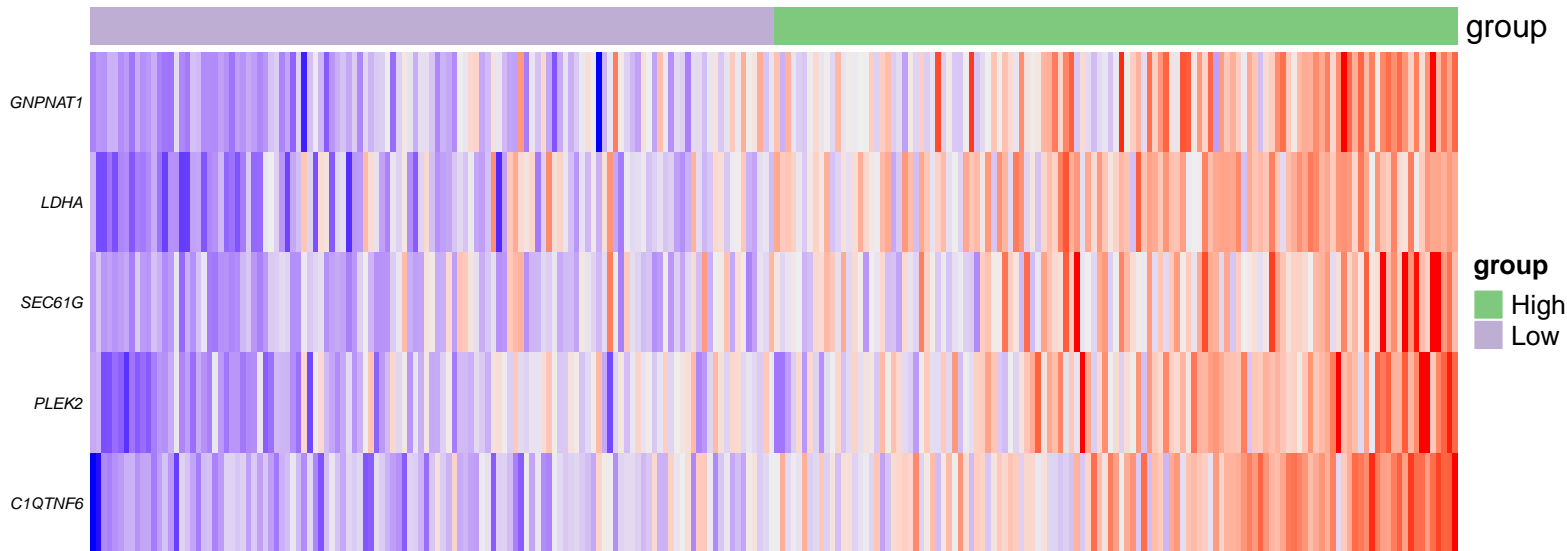

gse13213

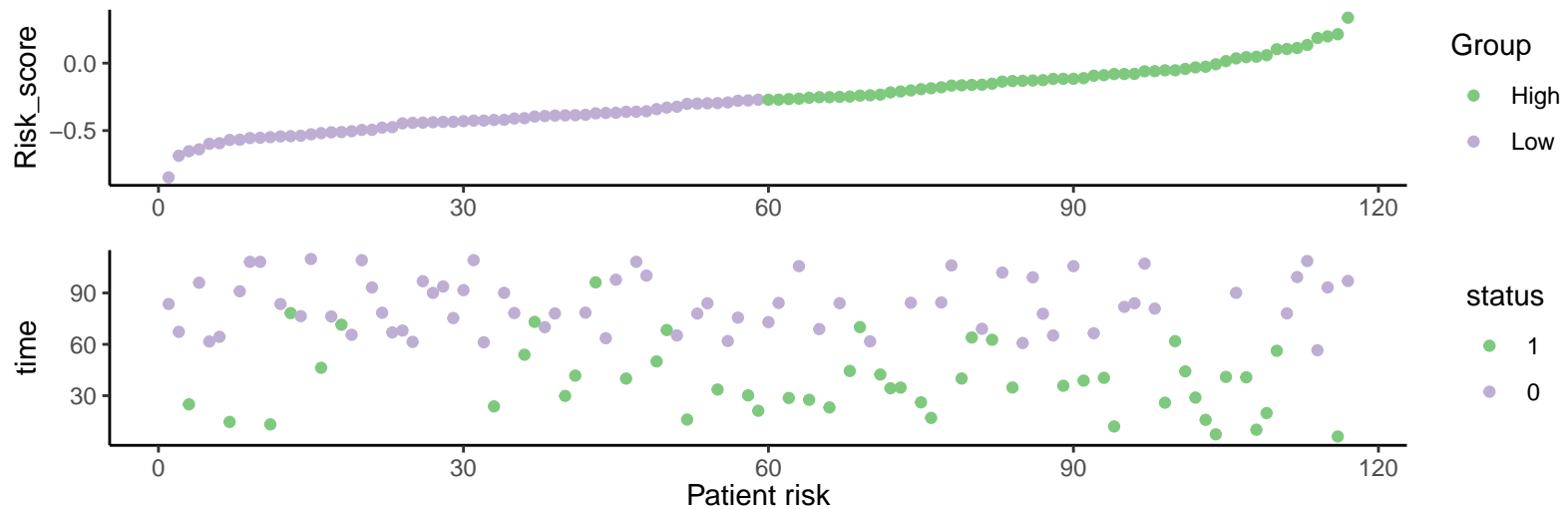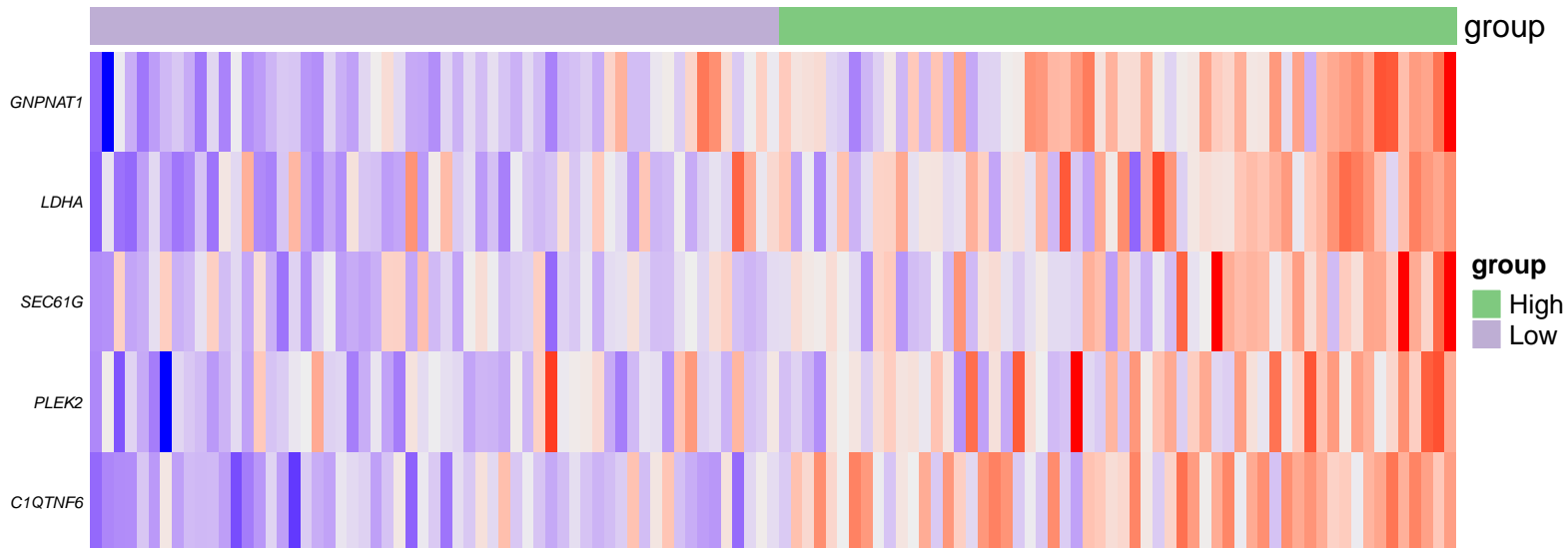

Supplement: Supplementary file 5 [file DataSheet6.PDF]

A

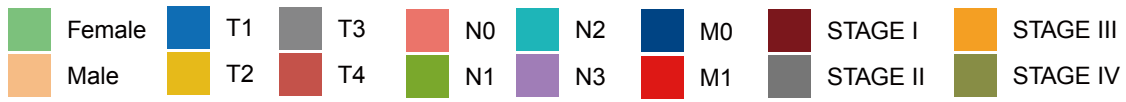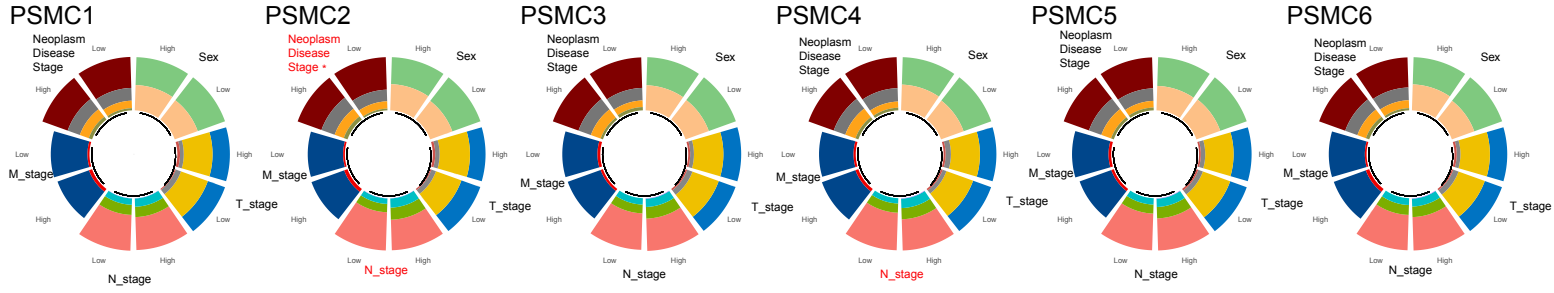

B

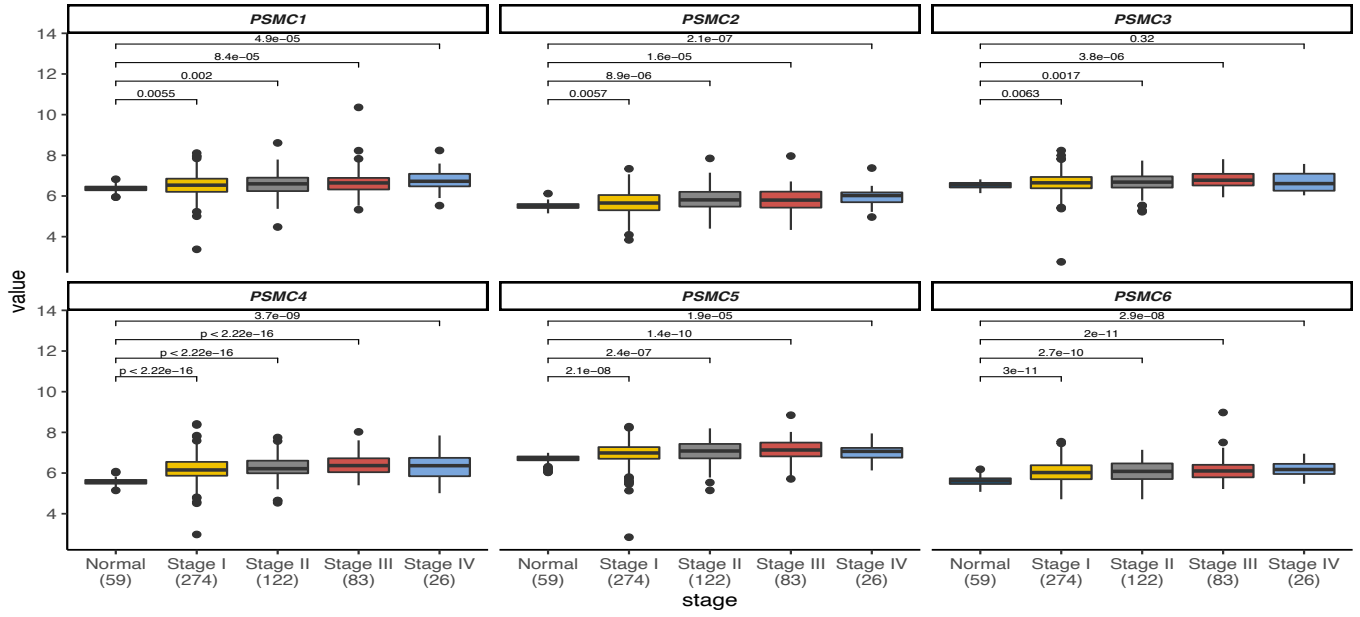

C

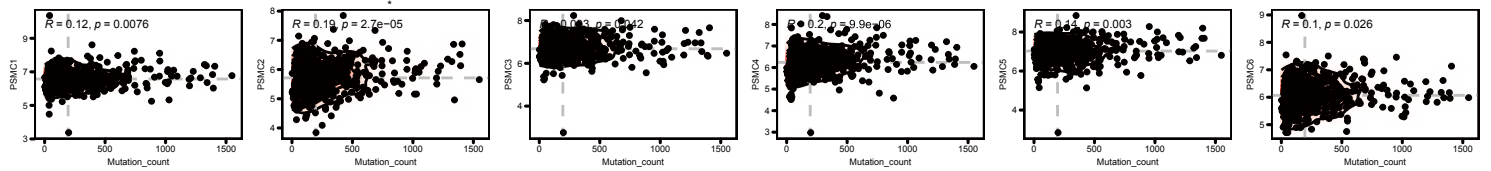

D

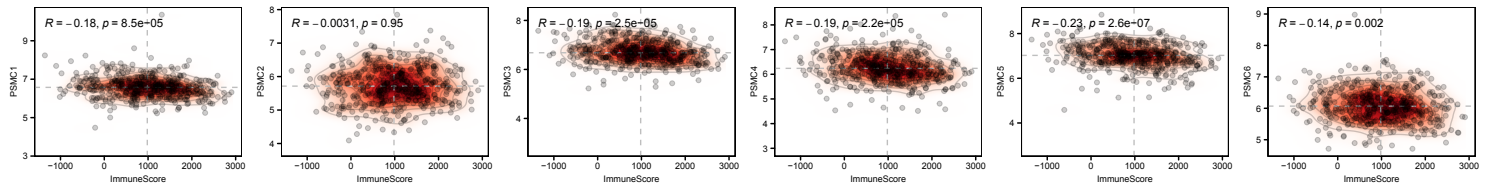

E

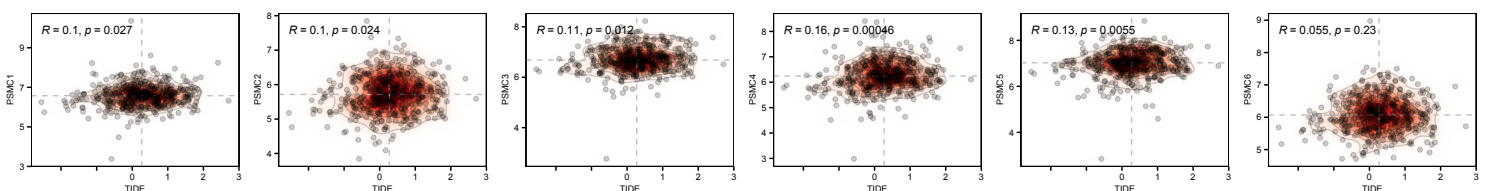

Supplement: Supplementary file 7 [file DataSheet3.PDF]

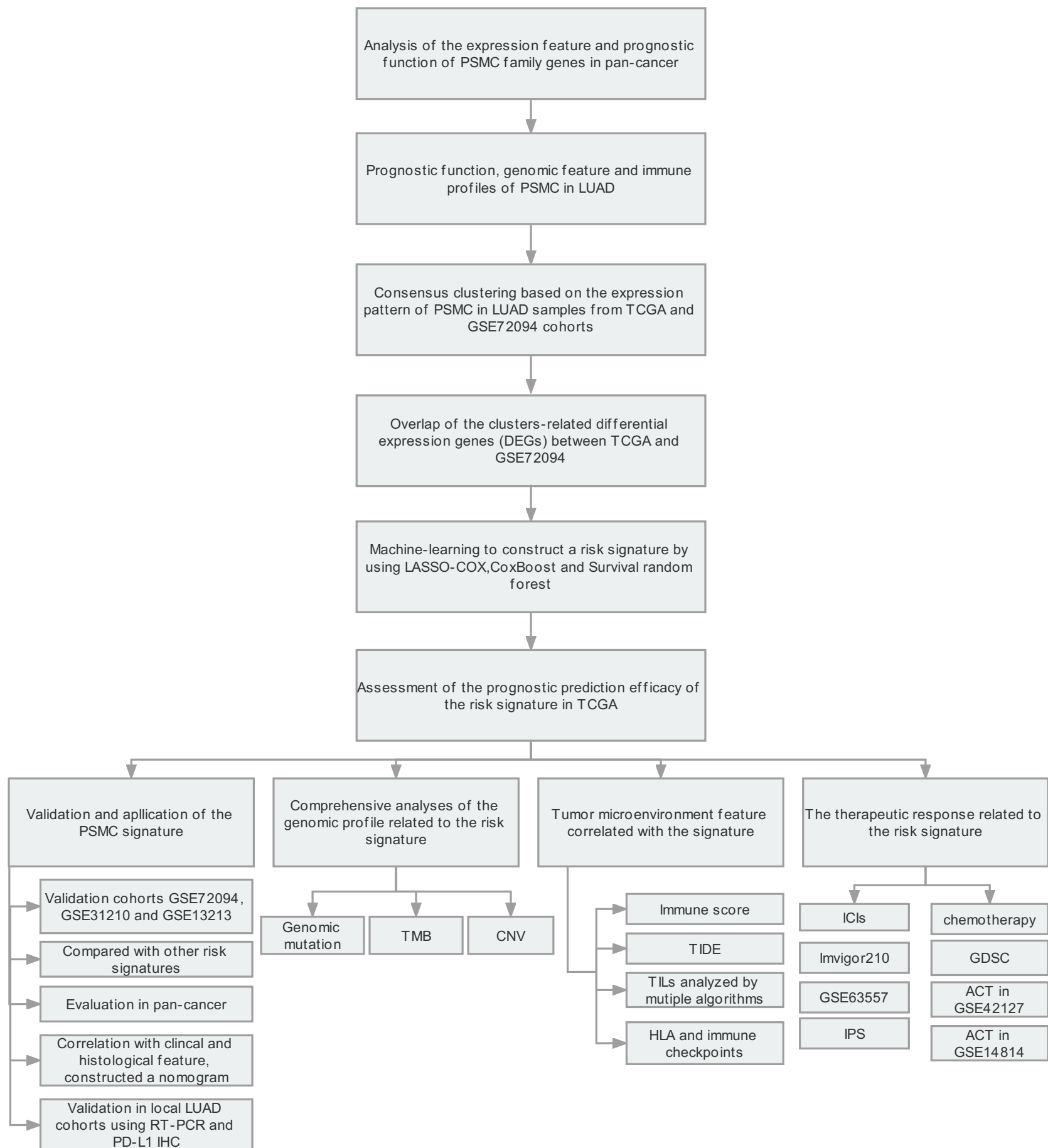

Supplement: Supplementary file 8 [file DataSheet1.PDF]

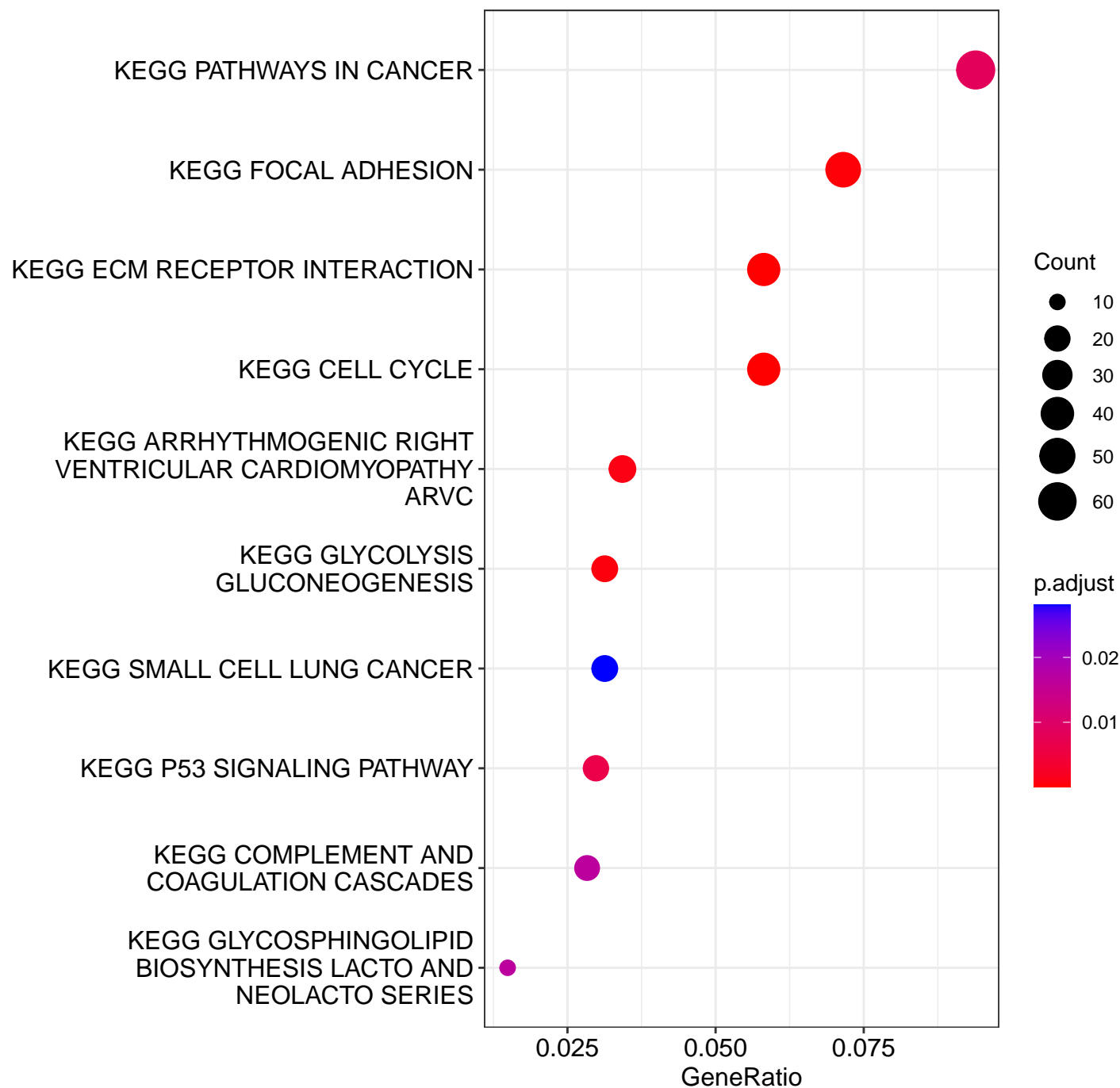

Supplement: Supplementary file 12 [file DataSheet8.PDF]
