# Supplementary material for: Pan-cancer analysis identifies proteasome 26S subunit, ATPase (PSMC) family genes, and related signatures associated with prognosis, immune profile, and therapeutic response in lung adenocarcinoma
Source: Front Genet. 2023 Jan 9;13:1017866. doi: 10.3389/fgene.2022.1017866 (PMC9868736; doi:10.3389/fgene.2022.1017866)
Supplement: Supplementary file 9 [file DataSheet5.PDF]

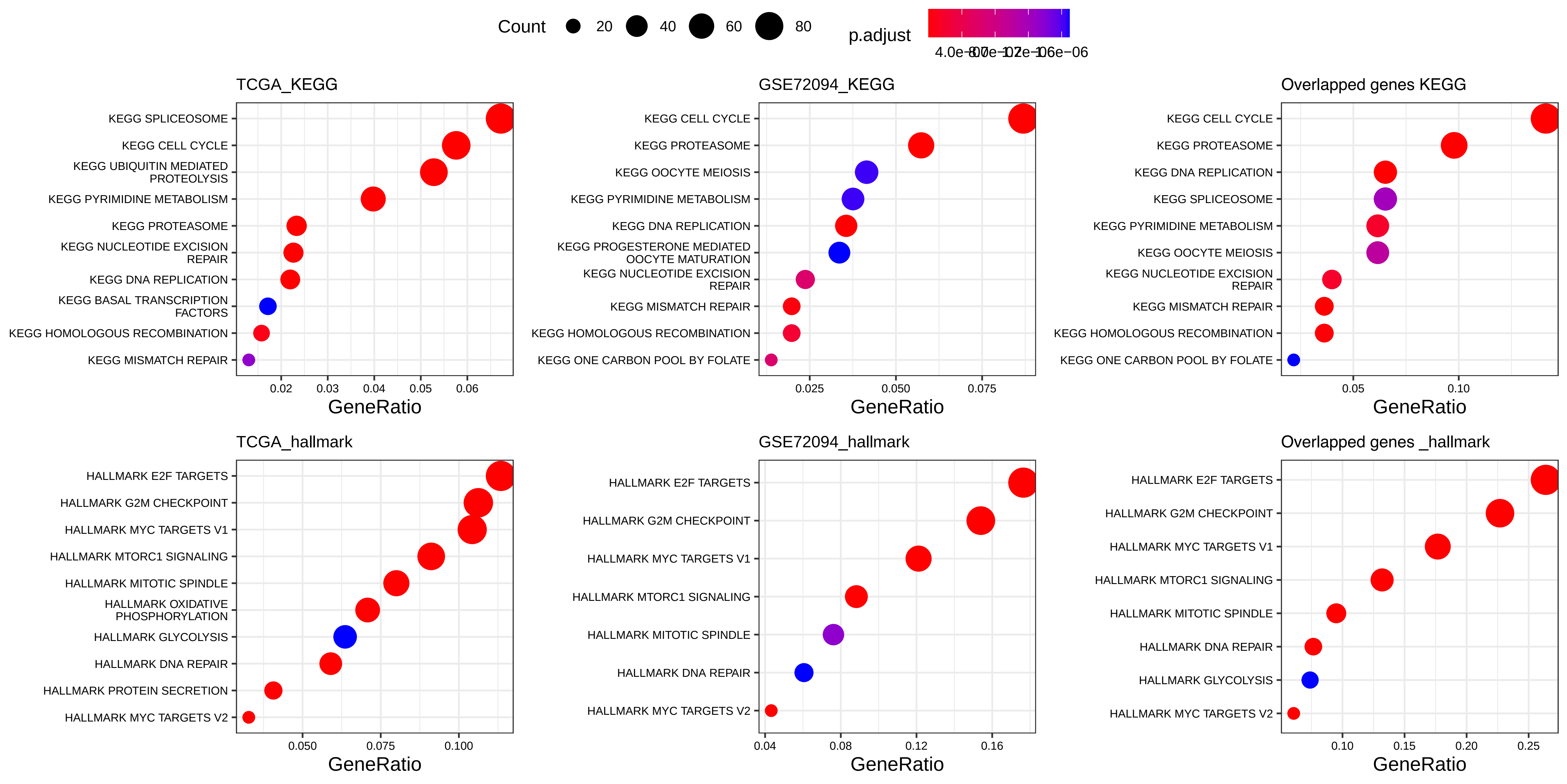
Count
20
40
60
80
p.adjust
TCGA_KEGG
KEGG SPLICEOSOME
KEGG CELL CYCLE
KEGG UBIQUITIN MEDIATED PROTEOLYSIS
KEGG PYRIMIDINE METABOLISM
KEGG PROTEASOME
KEGG NUCLEOTIDE EXCISION REPAIR
KEGG DNA REPLICATION
KEGG BASAL TRANSCRIPTION FACTORS
KEGG HOMOLOGOUS RECOMBINATION
KEGG MISMATCH REPAIR
0.02
0.03
0.04
0.05
0.06
GeneRatio
GSE72094_KEGG
KEGG CELL CYCLE
KEGG PROTEASOME
KEGG OOCYTE MEIOSIS
KEGG PYRIMIDINE METABOLISM
KEGG DNA REPLICATION
KEGG PROGESTERONE MEDIATED OOCYTE MATURATION
KEGG NUCLEOTIDE EXCISION REPAIR
KEGG MISMATCH REPAIR
KEGG HOMOLOGOUS RECOMBINATION
KEGG ONE CARBON POOL BY FOLATE
0.025
0.050
0.075
GeneRatio
Overlapped genes KEGG
KEGG CELL CYCLE
KEGG PROTEASOME
KEGG DNA REPLICATION
KEGG SPLICEOSOME
KEGG PYRIMIDINE METABOLISM
KEGG OOCYTE MEIOSIS
KEGG NUCLEOTIDE EXCISION REPAIR
KEGG MISMATCH REPAIR
KEGG HOMOLOGOUS RECOMBINATION
KEGG ONE CARBON POOL BY FOLATE
0.05
0.10
GeneRatio
TCGA_hallmark
HALLMARK E2F TARGETS
HALLMARK G2M CHECKPOINT
HALLMARK MYC TARGETS V1
HALLMARK MTORC1 SIGNALING
HALLMARK MITOTIC SPINDLE
HALLMARK OXIDATIVE PHOSPHORYLATION
HALLMARK GLYCOLYSIS
HALLMARK DNA REPAIR
HALLMARK PROTEIN SECRETION
HALLMARK MYC TARGETS V2
0.050
0.075
0.100
GeneRatio
GSE72094_hallmark
HALLMARK E2F TARGETS
HALLMARK G2M CHECKPOINT
HALLMARK MYC TARGETS V1
HALLMARK MTORC1 SIGNALING
HALLMARK MITOTIC SPINDLE
HALLMARK DNA REPAIR
HALLMARK MYC TARGETS V2
0.04
0.08
0.12
0.16
GeneRatio
Overlapped genes _hallmark
HALLMARK E2F TARGETS
HALLMARK G2M CHECKPOINT
HALLMARK MYC TARGETS V1
HALLMARK MTORC1 SIGNALING
HALLMARK MITOTIC SPINDLE
HALLMARK DNA REPAIR
HALLMARK GLYCOLYSIS
HALLMARK MYC TARGETS V2
0.10
0.15
0.20
0.25
GeneRatio
